# Proline pre-conditioning of cell monolayers increases post-thaw recovery and viability by distinct mechanisms to other osmolytes†‡

**DOI:** 10.1039/d1md00078k

**Published:** 2021-05-18

**Authors:** Trisha L. Bailey, Juan Ramon Hernandez-Fernaud, Matthew I. Gibson

**Affiliations:** Department of Chemistry, University of Warwick Gibbet Hill Road Coventry CV4 7AL UK m.i.gibson@warwick.ac.uk; Unidad de Investigación, Hospital Universitario de Canarias Calle Ofra s/n, La Cuesta La Laguna Tenerife Spain; Warwick Medical School, University of Warwick Gibbet Hill Road Coventry CV4 7AL UK

## Abstract

Cell cryopreservation is an essential tool for drug toxicity/function screening and transporting cell-based therapies, and is essential in most areas of biotechnology. There is a challenge, however, associated with the cryopreservation of cells in monolayer format (attached to tissue culture substrates) which gives far lower cell yields (<20% typically) compared to suspension freezing. Here we investigate the mechanisms by which the protective osmolyte l-proline enhances cell-monolayer cryopreservation. Pre-incubating A549 cells with proline, prior to cryopreservation in monolayers, increased post-thaw cell yields two-fold, and the recovered cells grow faster compared to cells cryopreserved using DMSO alone. Further increases in yield were achieved by adding polymeric ice recrystallization inhibitors, which gave limited benefit in the absence of proline. Mechanistic studies demonstrated a biochemical, rather than biophysical (*i.e.* not affecting ice growth) mode of action. It was observed that incubating cells with proline (before freezing) transiently reduced the growth rate of the cells, which was not seen with other osmolytes (betaine and alanine). Removal of proline led to rapid growth recovery, suggesting that proline pre-conditions the cells for cold stress, but with no impact on downstream cell function. Whole cell proteomics did not reveal a single pathway or protein target but rather cells appeared to be primed for a stress response in multiple directions, which together prepare the cells for freezing. These results support the use of proline alongside standard conditions to improve post-thaw recovery of cell monolayers, which is currently considered impractical. It also demonstrates that a chemical biology approach to discovering small molecule biochemical modulators of cryopreservation may be possible, to be used alongside traditional (solvent) based cryoprotectants.

## Abstract 2

Cell cryopreservation is an essential tool for transporting cell-based therapies, and is essential in most areas of biotechnology. Here proline pre-incubation prior to cell monolayer cryopreservation is explored, increasing post-thaw yields.

## Graphical abstract

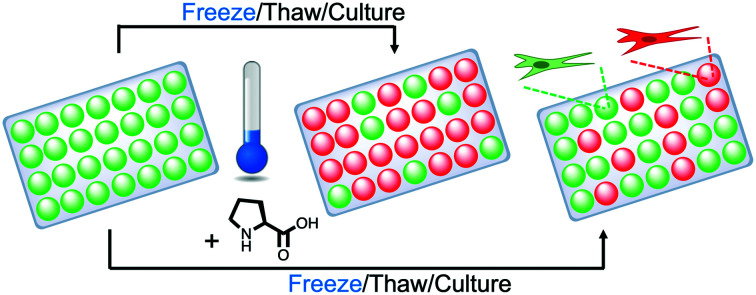

## Introduction

The storage, recovery, and manipulation of viable cells is the key component of all cell biology research, with primary cells being the most rare/valuable, and stem cells are currently emerging for many personalized medical treatments.^1,2^ Cell cryopreservation can give variable results with the phenotypic impact of frozen/thawed cells still unknown in many cases. Standard protocols use 5–10 wt% of the cryoprotective agent (CPA) dimethyl sulfoxide (DMSO), which is able to enter cells and reduce injury by moderating the increase in solute concentration during freezing.^3–5^ While suspension freezing in DMSO is highly successful, certain cells such as leukocytes (RAW 264.7) are highly-sensitive to DMSO.^6^ Additionally, there are some concerns with using DMSO due to its cytotoxicity at high concentrations and/or room temperature.^7–11^ Freezing with DMSO is the gold standard for suspension cell cryopreservation in nearly every research laboratory but the method does not work well for cell monolayers,^12^ typically resulting in only around 20–35% recovery, often even lower.^13,14^ For example, adherent human embryonic stem cells yield extremely low survival rates of <5%.^12,13^ The ability to cryopreserve monolayered cells would facilitate drug development by providing phenotypically identical cells for assays, reduce the total resource needed for cell culture (*e.g.* cells are already in format for assay) as well as providing insights into the cryopreservation of more complex biological material such as spheroids or tissues.

According to Mazur's two-factor hypothesis, key challenges in cryopreservation are ice formation and osmotic stress due to the addition of cryoprotective agents and the increasing concentration of solutes in the remaining water phase during the freezing process.^15,16^ Intracellular ice can form when the cooling rate is intermediately high and the cell cannot maintain osmotic equilibrium with the environment, and is almost always lethal.^17,18^ Controlled-rate freezing ensures movement of water across the plasma membrane in order for osmotic dehydration to reach equilibrium with intracellular and extracellular contents to prevent intracellular ice.^19,20^ During thawing, ice recrystallization can damage the cells by both mechanical and osmotic stress, and controlling this process by using ice recrystallization inhibitors (IRIs) has been shown to result in enhanced post-thaw recovery.^21,22^ Antifreeze (glyco)proteins (AF(G)Ps) are potent IRIs and can reduce damage to cell membranes,^23–25^ however, AF(G)Ps are unsuitable for cryopreservation applications due to their potential toxicity/immunogenicity, their secondary effect of dynamic ice shaping (DIS),^21^ and their low availability/expense. Considering this, there is significant interest in the discovery of AF(G)P mimics.^21,26–28^

The most studied IRI active polymer is poly(vinyl alcohol) (PVA), due to its potent IRI, low toxicity, and commercial availability.^24,29,30^ PVA gives some benefit to the cryopreservation of cells in suspension,^31–33^ but has not been explored for cell monolayer cryopreservation, although polyproline (a weak IRI) was reported to enhance monolayer recovery when used in conjunction with the protective osmolyte proline^34^ and has been applied to oocytes also.^35^ Without addition of proline, there was limited benefit of the IRI-active polymer, but the functional role of this proline incubation is not fully understood.

Cells almost universally respond to the stress of long-term hyperosmolality, such as during freezing, by accumulating compatible organic osmolytes.^36^ The major osmolytes in water-stressed eukaryotes include metabolic products such as glycerol and sucrose, amino acids (plus derivatives such as taurine and β-alanine), urea, and methylamines (*e.g.* tri-methylamine-*N*-oxide).^37^ The most widely studied ‘compatible’ solutes are the *N*-substituted amino acids, particularly betaine and proline.^38^ Betaine's osmoprotection has been widely observed, such as in the dogfish shark *Squalus acanthias* promoting betaine efflux during hypotonic conditions.^39^ Betaine reduces leakage from frozen multilamellar liposomes,^40^ and can enhance the vitrification of mammalian cells in suspension.^41^ Proline is membrane permeable regardless of pH,^42^ and transfer can occur by a range of processes.^43^ Proline has been implicated as a survival factor that protects the cell against apoptosis and maintains the progression of the cell cycle through a signal recognition function of the transporters that may take part in the control of cell cycle progression and programmed cell death.^44^ Proline has been used with success to cryopreserve mouse oocytes,^45^ vitrify red blood cells (RBCs) and mammalian cells,^46^ induce freeze-tolerance in fruit fly larvae,^47^ and has shown protection for mammalian cell monolayers when used alongside trehalose.^14^

Considering the above, this study explores the use of proline for the cryopreservation of adherent cell monolayers in comparison to other protective osmolytes. It is shown that incubation of cell monolayers with proline, in advance of cryopreservation, leads to increases in post-thaw recovery. Addition of polymeric IRIs further increased the post-thaw cell yield, but only when used with proline, not without. Evidence is presented that proline prepares the cells for freezing by a temporary slowing of cellular growth which was not seen for other osmolytes and may explain its beneficial role in cell monolayer cryopreservation.

## Experimental section

### Cell culture

#### Reagents and solution preparation

PVA (*M*_w_ 9000–10 000, 80% hydrolysed), proline, alanine and betaine were obtained from Sigma Aldrich Co Ltd, (Irvine, UK). Solutions for cell experiments were prepared by dissolving the individual compounds in base cell media supplemented with 10% FBS and 1× PSA (solutions used as CPAs did not contain PSA) and sterile filtering prior to use.

#### A549 cell culture

Human Caucasian lung carcinoma cells (A549) were obtained from the European Collection of Authenticated Cell Cultures (ECACC) (Salisbury, UK) and grown in 175 cm^2^ cell culture Nunc flasks (Corning Incorporated, Corning, NY). Standard cell culture medium was composed of Ham's F-12K (Kaighn's) Medium (F-12K) (Gibco, Paisley, UK) supplemented with 10% USA-origin fetal bovine serum (FBS) purchased from Sigma Aldrich (Dorset, UK), 100 units per mL penicillin, 100 μg mL^−1^ streptomycin, and 250 ng mL^−1^ amphotericin B (PSA) (HyClone, Cramlington, UK). F-12K contains 0.2 mM alanine, 0.6 mM proline and has an osmolarity of 275 to 357 mOsm kg^−1^. A549 cells were maintained in a humidified atmosphere of 5% CO_2_ and 95% air at 37 °C and the culture medium was renewed every 3–4 days. The cells were subcultured every 7 days or before reaching 90% confluency. To subculture, cells were dissociated using 0.25% trypsin plus 1 mM EDTA in balanced salt solution (Gibco) and reseeded at 1.87 × 10^5^ cells per 175 cm^2^ cell culture flasks.

#### Cryopreservation of cell monolayers

Methods adapted from Bailey *et al.*^14^ Cells to be frozen in monolayer format were seeded at 0.4 × 10^6^ (A549) or 0.5 × 10^6^ (Neuro-2a, MC-3T3) cells per well in 500 μL of cell culture medium in 24-well plates (Corning Incorporated, Corning, NY). Plates had a total available volume of 3.4 mL with an approximate growth area of 1.9 cm^2^, no coverslips were used and plates were used with the accompanying lid. Cells were allowed to attach to the entire free surface of the bottom of the well and formed a confluent layer not greater in height than one cell. Before experimental treatments, cells were allowed to attach for 2 h to the plates in a humidified atmosphere of 5% CO_2_ and 95% air at 37 °C. The medium was exchanged against medium that was or was not supplemented with solutes as indicated in the figures. Control cells received no additional solutes and experimental cells were incubated in indicated solutions for 24 h in a humidified atmosphere of 5% CO_2_ and 95% air at 37 °C. Following the incubation period, the culture medium was removed and cells were exposed for 10 min at room temperature to different concentrations of solutes dissolved in base media supplemented with 10% FBS and indicated concentrations of DMSO. After 10 min, the freezing solutions were removed and the plate placed inside a CoolCell® MP plate (BioCision, LLC, Larkspur, CA), transferred to a −80 °C freezer and frozen at a rate of 1 °C min^−1^. After 24 h at −80 °C, cells were rapidly thawed by addition of 500 μL complete cell culture medium warmed to 37 °C. Cells were placed in a humidified atmosphere for 24 h and then dissociated using 0.25% trypsin plus 1 mM EDTA in balanced salt solution. The number of viable cells was then determined by counting with a hemocytometer (Sigma Aldrich) at room temperature after 1 : 1 dilution of the sample with 0.4% trypan blue solution (Sigma Aldrich). The initial cell medium was discarded such that any non-attached cells were not included in the assessment. The percentage of recovered cells was calculated by dividing the number of cells with intact membranes after freezing and thawing by the number of cells present prior to freezing (*i.e.* after application of pre-treatments), multiplied by 100.

#### Post-freezing cell viability assay

Cells were seeded post-thaw at 12.5 × 10^3^ per well in 6-well plates in 2 mL of complete cell media. Cells were placed in a humidified atmosphere of 5% CO_2_ and 95% air at 37 °C and allowed to grow, with counts starting on day two and ending on day six. Cell media was renewed on day three. Cells were dissociated using 0.25% trypsin plus 1 mM EDTA in balanced salt solution. The number of viable cells was then determined by counting with a hemocytometer at room temperature after 1 : 1 dilution of the sample with 0.4% trypan blue solution. Fold increase of cells was calculated by dividing the number of cells with intact cell membranes by the number of cells initially plated.

#### Incubation growth assay

Cells were seeded at 12.5 × 10^3^ per well in 6-well plates (Thermo Fisher) in 2 mL of complete cell media or in the indicated solutions. Cells were placed in a humidified atmosphere of 5% CO_2_ and 95% air at 37 °C and allowed to grow, with counts starting on day two and ending on day six. Cell media was renewed on day three. Cells were dissociated using 0.25% trypsin plus 1 mM EDTA in balanced salt solution. The number of viable cells was then determined by counting with a hemocytometer (Sigma Aldrich) at room temperature after 1 : 1 dilution of the sample with 0.4% trypan blue solution (Sigma Aldrich). Fold increase of cells was calculated by dividing the number of cells with intact cell membranes by the number of cells initially plated.

#### Incubation recovery growth assay

Cells were seeded at 12.5 × 10^3^ per well in 6-well plates (Thermo Fisher) in 2 mL of complete cell media or in the indicated solutions. Cells were placed in a humidified atmosphere of 5% CO_2_ and 95% air at 37 °C and allowed to grow, with counts starting on day four and ending on day six. Cell media was renewed on day three and cells incubated in solutes were switched back to complete cell media. Cells were dissociated using 0.25% trypsin plus 1 mM EDTA in balanced salt solution. The number of viable cells was then determined by counting with a hemocytometer (Sigma Aldrich) at room temperature after 1 : 1 dilution of the sample with 0.4% trypan blue solution (Sigma Aldrich). Fold increase of cells was calculated by dividing the number of cells with intact cell membranes by the average number of cells on day three from the incubation growth assay.

#### Osmolyte incubation calcein/ethidium homodimer-1 uptake

A549 cells were seeded at 4 × 10^5^ cells per well in 500 μL of cell culture medium in 24-well plates. Cells were incubated with osmolyte solutions for 24 h in a humidified atmosphere of 5% CO_2_ and 95% air at 37 °C. Following the 24 h incubation, cells were incubated with 0.3 μM calcein-AM (ThermoFisher) and 10 μM ethidium homodimer-1 (ThermoFisher) in PBS. Cells were incubated at room temperature for 45 min in the dark. Solution was removed and wells were washed twice with PBS. Plate was read using the BioTex plate reader at 494/517 nm and 528/617 nm. Plate was then imaged with a CKX41 microscope with pE-300-W LED illumination, XC30 camera, and processed using CellSens software.

#### Post-freeze membrane calcein/ethidium homodimer-1 uptake

A549 cells were cryopreserved as monolayers as previously indicated. Following the 24 h post-thaw incubation, cells were incubated with 0.3 μM calcein and 10 μM ethidium homodimer-1 in PBS. Cells were incubated at room temperature for 45 min in the dark. Solution was removed and wells were washed twice with PBS. Plate was read using the BioTex plate reader at 494/517 nm and 528/617 nm. Plate was then imaged with a CKX41 microscope with pE-300-W LED illumination, XC30 camera, and processed using CellSens software.

### Physical and analytical methods

#### Differential scanning calorimetry

Samples were prepared by weighing standard 40 μL aluminium crucibles (Mettler Toledo, Leicestershire, UK) and adding 15 μL of analyte before sealing and reweighing in order to quantify the exact mass of sample. Each sample was then transferred to a liquid nitrogen cooled DSC 1 STAR® system (Mettler Toledo, Leicestershire, UK) differential scanning calorimeter. The mass of the aluminium crucible and sample mass was input into the complimentary STARe thermal analysis software to retain a digital record and aid analysis. Each DSC sample was individually cooled from +25 °C to −150 °C at a rate of 10 °C min^−1^ whilst concurrently monitoring the heat flow (mW) of the system to detect any endothermic or exothermic transitions. When samples were cooled to −150 °C, each sample was held for 10 min and then warmed at a rate of 10 °C min^−1^ from −150 °C to +25 °C. Raw data from each experiment was exported and plotted in R (R Foundation for Statistical Computing, Vienna, Austria) and individual peaks highlighted for comparison using the STARe thermal analysis software built-in modelling functions for linear curve fitting and area under the curve when required.

#### Ice recrystallization ‘splat’ assays

A 10 μL droplet of compound in the indicated solution was dropped using a Hamilton gastight 1750 syringe (Hamilton Bonaduz AG, GR, Switzerland) from a fixed height of 1.4 m onto a 22 × 22 mm no.1 glass cover slip (Fisher Scientific UK Ltd, Leicestershire, UK) placed on a CO_2(s)_ cooled aluminium plate (∼−70 °C). The droplet froze instantly upon impact with the plate forming a thin wafer of ice. This wafer was then placed on a liquid nitrogen cooled BCS196 cryostage (Linkam Scientific, Surrey, UK) and held at −8 °C to anneal for 30 min using the LNP96 cooling system (Linkam Scientific). Photographs were taken using a Canon EOS 500D SLR digital camera (Canon (UK) Ltd, Surrey, UK) after 0 and 30 minutes coupled to an Olympus CX41 microscope (Olympus, Southend-on-Sea, UK) equipped with UIS-2 20×/0.45/∞/0–2/FN22, UIS-2 4×/0.1/∞/–/FN22 and UIS-2 10×/0.2/∞/–/FN22 lenses (Olympus Ltd, Essex, UK) through cross polarisers. The number of crystals in the image was counted using ImageJ (version 1.52a)^48^ and the area of the field of view divided by the number of crystals gave the average crystal size per wafer.

## Results and discussion

**Fig. 1:**
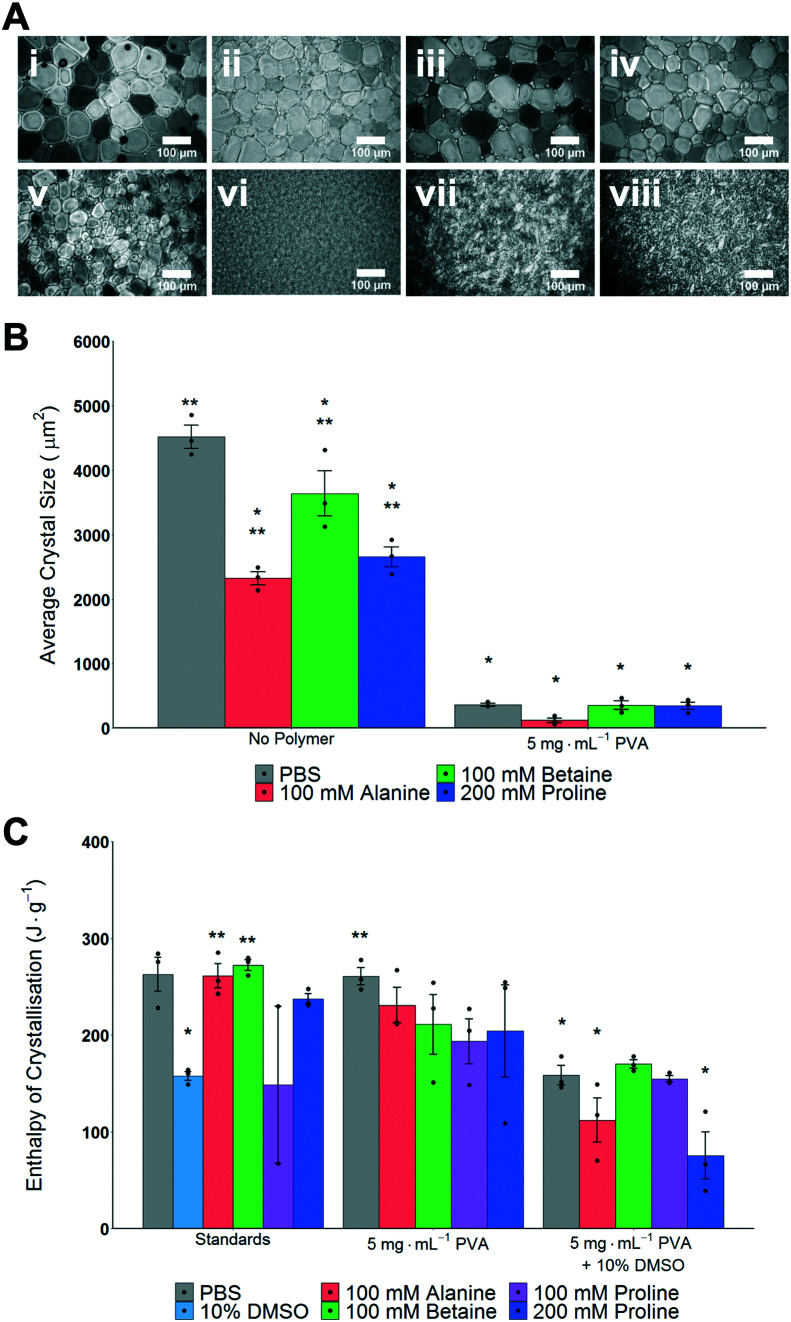
Impact of cryoprotectants on ice formation and growth. A) Example ice wafers from ‘splat assay’ following annealing for 30 minutes; i) PBS, ii) 100 mM alanine in PBS, iii) 100 mM betaine in PBS, iv) 200 mM proline in PBS, v) 5 mg mL^−1^ PVA in PBS, vi) 100 mM alanine/5 mg mL^−1^ PVA in PBS, vii) 100 mM betaine/5 mg mL^−1^ PVA in PBS, viii) 200 mM proline/5 mg mL^−1^ PVA in PBS. Scale bar = 100 μm. B) Average ice crystal sizes determined from splat test. *P < 0.0001 from PBS. **P < 0.0001 from 5 mg mL^−1^ PVA in PBS. Note: average crystal size is not reported as a % of PBS (which is standard) to allow for statistical comparisons. All error bars represent ± SEM of 3 independent experiments, dots indicate individual data points. C) Average enthalpy of ice crystallisation determined by DSC. *P < 0.0001 from PBS. **P < 0.001 from 10% DMSO (100 mM proline (standards) *N* = 2 and was not included in statistical assessment). All error bars represent ± SEM of 3 independent experiments, dots indicate individual data points.

The main aim of this study was to explore the use, and mechanisms, of proline as an additive to enhance the cryopreservation of cell monolayers, in comparison to other osmolytes. A second objective was to explore the synergy of proline with ice recrystallization inhibitors (IRIs) which may also aid cell monolayer cryopreservation. Betaine and alanine were chosen as control osmolytes to be used throughout this study. To evaluate if ice recrystallization inhibition (*i.e.* preventing ice crystals from growth) occurs in the presence of osmolytes the ‘splat assay’ was used.^24,49^ This was important, as several studies have shown that the exact ions in solution can impact IRI activity, and any subsequent cryopreservation results can only be interpreted if this effect is known.^50,51^ The assay involves rapid nucleation of small (<10 μm) ice crystals followed by annealing at −8 °C for 30 minutes. After this time, the wafers were imaged and the size of the ice crystals was assessed; smaller crystals mean more IRI activity, Fig. 1A. For the osmolyte solutions, significantly smaller crystal sizes than PBS alone were observed, yet all also had significantly larger crystal sizes than 5 mg mL^−1^ PVA in PBS. The data is summarized in Fig. 1B, as average crystal size. Note, mean grain size (MGS), the standard measure^52^ is not used as the large differences between conditions makes this comparison challenging. It is important to observe that any additive at sufficiently high concentration will slow ice growth, and under these conditions the osmolytes alone would not be considered IRI active.^52^

As high (>100 mM) concentrations of osmolytes will be used in the cryopreservation studies, it was also essential to investigate the impact osmolytes have on total ice formation during cryopreservation conditions. Fig. 1C shows the total enthalpy of crystallization (which is proportional to the total volume of ice formed) for each osmolyte (in PBS), 10% DMSO and with/without PVA as determined by differential scanning calorimetry. When DMSO is used there is an expected decrease in the enthalpy of crystallisation,^53^ but significant ice is still forming, which confirms the conditions employed here are not leading to vitrification (an alternative method of cryopreservation). Full DSC traces for melting/freezing are shown in Fig. S1 and S2.‡ These results demonstrate that the osmolytes, at the concentrations used, do not significantly impact the physical process of ice formation and growth, which is essential to enable their biochemical roles (below) to be investigated.

Following the above studies on ice formation/growth, A549 (adenocarcinomic human alveolar basal epithelial) cells were employed to test the cytotoxicity of each component, after 24 hours exposure. PVA (polymeric ice recrystallization inhibitor) led to a small reduction in metabolic activity (Fig. S3‡), and there was no significant reduction for any of the osmolytes (proline, betaine, alanine (Fig. S4‡)), and hence cryopreservation studies could be conducted.

**Fig. 2:**
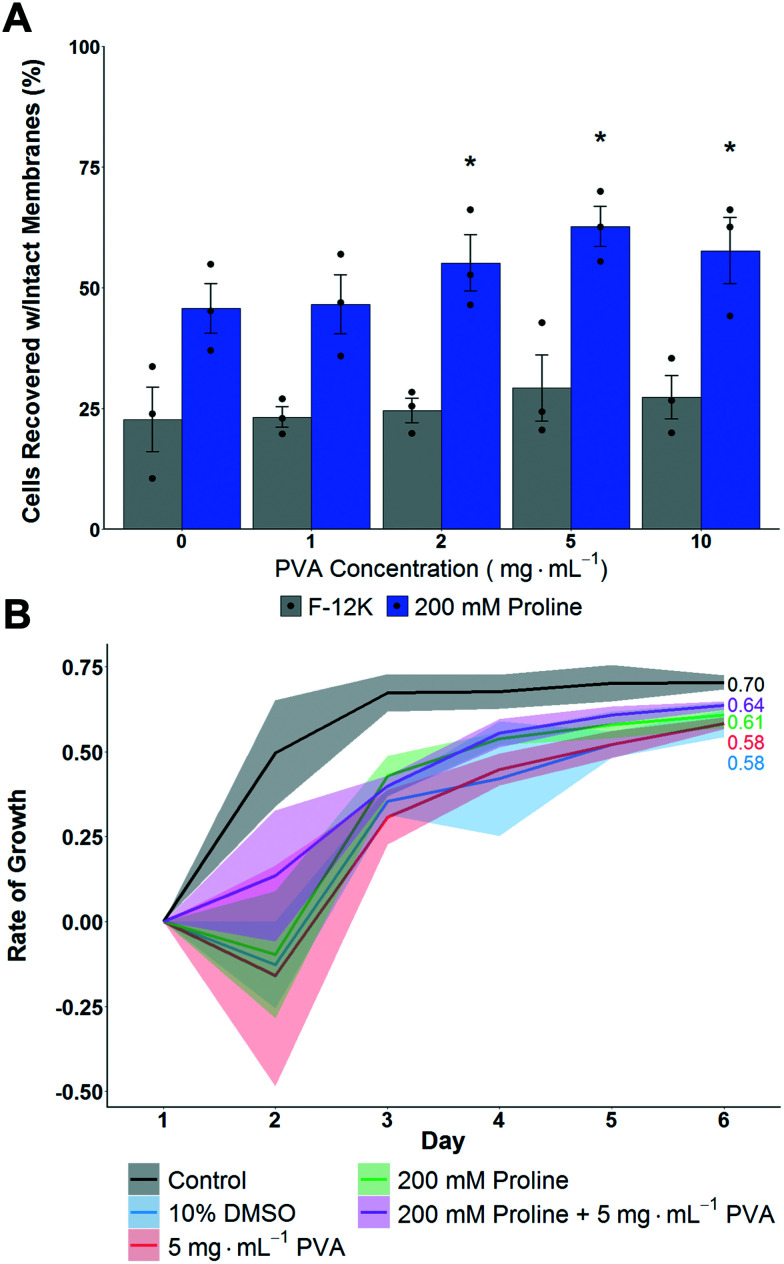
Impact of proline on post-thaw recovery and growth of A549 monolayers after cryopreserving at −80 °C. A) Total cell recovery 24 hours post thaw, determined *via* trypan blue assay. Error bars represent ± SEM of 3 independent experiments with two nested replicates. *P < 0.00001 from control (0 mg mL^−1^ in F-12K). B) Growth rate of A549 cell monolayers post-thaw. Shaded area represents standard error of 3 independent experiments. *R*^2^ values are indicated.

A549 cell monolayers were evaluated for cryopreservation (using 10 vol% DMSO) with and without proline pre-incubation (for 24 hours to allow time for pre-conditioning^14,34^) and with variable concentrations of PVA as the IRI active agent, Fig. 2A. This was to determine the additive effect of proline when supplemented into standard cryopreservation conditions. It is crucial to note at this point that excess CPA solutions were removed before freezing, thus allowing thawing by addition of pre-warmed media, which also diluted any residual cryoprotectants while ensuring a rapid thawing process. Cells were allowed to recover for 24 hours post-thaw before assessment of recovery. This is important as shorter post-thaw times lead to false positives if cells are evaluated before apoptotic processes have had a chance to complete.^13,54^ Therefore the number presented is the number of cells after 24 hours, relative to those prior to freezing, and hence includes some time for potential cell growth, but remains a robust measure to avoid over-estimation if shorter times to assay are used.

Cryopreservation of A549 cell monolayers in 10% DMSO alone lead to <25% cell recovery, compared to >60% which is achieved in suspension cryopreservation of A549 cells,^55^ highlighting the significant challenge of directly cryopreserving cells in this format and the need for disruptive technologies. In contrast to the DMSO only conditions, pre-incubation with proline for 24 hours increased the post-thaw recovery to ∼50%, which is a remarkable increase and in line with previous observations of proline-mediated cryopreservation.^14^ Additional pre-incubation periods were not evaluated so the potential for further optimisation exists. Addition of PVA, to reduce ice recrystallization, further increased recovery to 60% recovery using 2–5 mg mL^−1^. Higher concentrations of PVA had no further benefit, as has been previously reported.^21,31^ Interestingly, without the proline pre-incubation, PVA did not have significantly positive effects on post-thaw recovery, supporting a hypothesis that ice recrystallization is a problem in cell monolayer storage, but that other damage mechanisms are more significant and must be mitigated first.

To gain further insight, the thawed A549 cells were isolated and re-plated at 12.5 × 10^3^ cells per well and their growth rate measured over 6 days. Fig. 2B shows that cells that had been pre-incubated with proline lead to faster growth rates compared to cells without. This shows that not only are more cells recovered, but that they are more viable due to the action of proline. In contrast, PVA increased the number of cells but had no impact on cell growth rate, and hence shows the polymer only has biophysical, not biochemical, function and shows synergistic protective mechanisms are in action. Two other cells lines were also explored, (MC-3T3 and Neuro-2a) where smaller effects were observed which may be due to specific cell differences including size but also their response to hypotonic conditions and contact inhibition (Fig. S5‡). This observation is crucial to highlight how the cell type and format (suspension verses monolayer, freezing rates, *etc.*) all have major impacts on cryopreservation outcomes and suggests there may not be ‘one size fits all’ solutions.

**Fig. 3:**
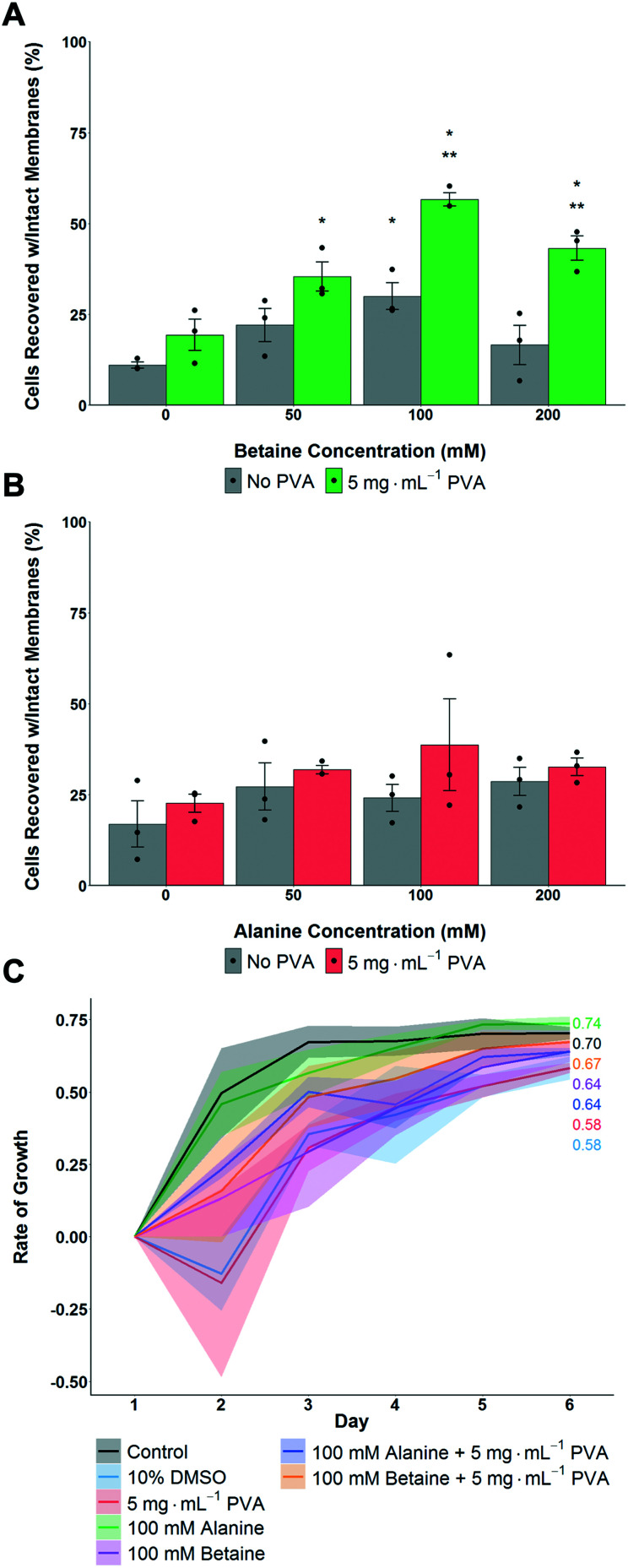
Impact of alanine and betaine on post-thaw recovery and growth of A549 monolayers after cryopreservation at −80 °C. A) Total cell recovery 24 hours post thaw with betaine as osmolyte, recovery determined *via* trypan blue assay. *P < 0.00001 from control (0 mg mL^−1^) with no PVA, **P < 0.0001 from control with 5 mg mL^−1^ PVA. B) Total cell recovery 24 hours post thaw with alanine as osmolyte, recovery determined *via* trypan blue assay. Error bars represent ± SEM of 3 independent experiments with two nested replicates, dots indicate individual data points. C) Post-thaw growth rates of A549 cell monolayers. Shaded area represents standard error of 3 independent experiments (100 mM alanine *N* = 2).

The above data shows that proline had a significant benefit for monolayer cryopreservation, and that recovered cells grew faster, at a rate closer to healthy non-frozen cells. To understand if proline's activity was simply due to it being an osmolyte, or if it had (for example) pre-conditioning effects,^56^ betaine and alanine were used as control osmolytes. A549 cells were pre-incubated with the osmolytes for 24 hours, then cryopreserved with 5 mg mL^−1^ PVA + 10% DMSO. Post thaw, the number of cells was determined after 24 hours culture. In the case of betaine, maximum cell recovery was observed with 100 mM, with PVA giving some benefit to cell recovery at all betaine concentrations (Fig. 3A). Alanine showed no significant increase in post-thaw recovery compared to control cells (Fig. 3B). All cells frozen with betaine or alanine, with or without PVA, grew faster post-freeze than cells frozen with only 10% DMSO (Fig. 3C). Betaine's osmoprotection has been previously studied,^41^ and attributed to the osmoregulated betaine/GABA transporter (BGT1) which couples the transport of betaine to Cl^−^ and Na^+^ across the cell membrane.^57,58^ Renomedullary cells grown in hyperosmotic media contained higher concentrations of betaine than cells grown in isotonic media^59^ and simian-virus-40-transformed Balb/c 3T3 (SV-3T3) cells were resistant to osmotic stress, which typically resulted in inhibition of protein synthesis and decreased proliferation, when treated exogenously with betaine.^60^ Taken together this data shows that proline and betaine have preferential roles in cryopreservation, as this is not simply an effect of added solutes or osmolarity, and are potent additives for cell monolayer cryopreservation. It is, however, not clear if betaine and proline afford their protection *via* the same mechanisms.

**Fig. 4:**
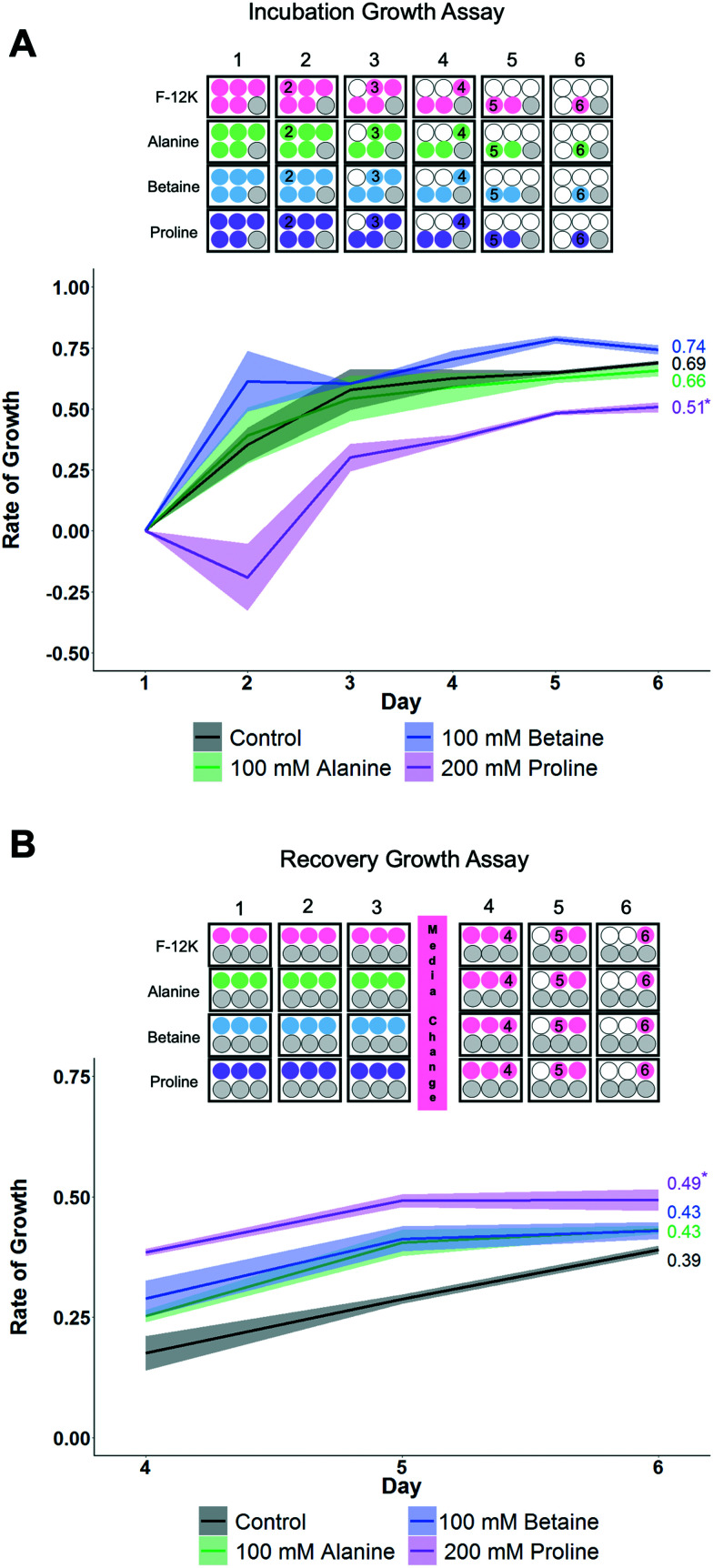
Impact of osmolytes on non-cryopreserved A549 cell monolayers. A) Growth rates during incubation with F-12K or osmolyte for 6 days. *P < 0.0002 from control. B) Growth rates after incubation with osmolyte for 3 days, then replacing media with F-12K media for 3 days (hence data starts on day 4). *P < 0.02 from control. Shaded area represents standard error of 3 independent experiments. Plate maps show experimental design, where cells are seeded, what treatment they received, and the day when cells are removed (for counting) indicated.

To gain more insight into how each osmolyte functions, growth curves for the cells were monitored as this provides a more rigorous evaluation of health than single point viability measures. Two sets of conditions were evaluated here (Fig. 4) using the concentrations of each osmolyte which gave maximal cell recovery (from Fig. 2 and 3). Firstly, cells were cultured over 6 days in the presence of osmolytes to allow their effects on overall cell growth to be monitored. This experiment is distinct from cytotoxicity assays, in that cells are seeded at low densities so they have space to propagate. Secondly, cells were incubated with osmolytes for 3 days, then exchanged for cell media, and allowed to recover for 3 more days.

When cells were cultured for 6 days in the presence of the osmolytes (at their optimised concentration), proline showed suppression of the growth rate compared to control cells and other osmolytes, which would imply proline has a growth-limiting impact on the cells (Fig. 4A). Next, the cells were incubated with the osmolytes for 3 days, and then the media replaced with standard (no osmolyte) growth media. The proline-treated cells showed a higher fold change in growth rate than other conditions, including control cells, and while the *R*^2^ value is lower than control values, it does trend upwards (Fig. 4B). This data shows that proline causes a short-term down-regulation in proliferation, which is rapidly recovered when proline is removed. P493 (B lymphoma) cells incubated in proline have been shown to have growth inhibition, and thus, resulted in the proline biosynthesis (PB) pathway being down-regulated with a downstream effect on the glycolytic pathway.^61^ This suggests that the lower proliferation rate seen here may not be the proline itself, but rather the effects of the PB pathway not running due to an increase in the available free proline. This down-regulation in growth may be partially responsible for the cryoprotection seen with proline, as metabolic depression and cell stasis are often prerequisites to survival for organisms with natural adaptations to desiccation, freezing temperatures, and anoxia.^62^ For example, pretreatment with 5-aminoimidazole-4-carboxamide-1-b-d-ribofuranoside (AICAR) can increase cell yields post-thaw, potentially due to metabolic down-regulation (*e.g.* diapause^56^).

**Fig. 5:**
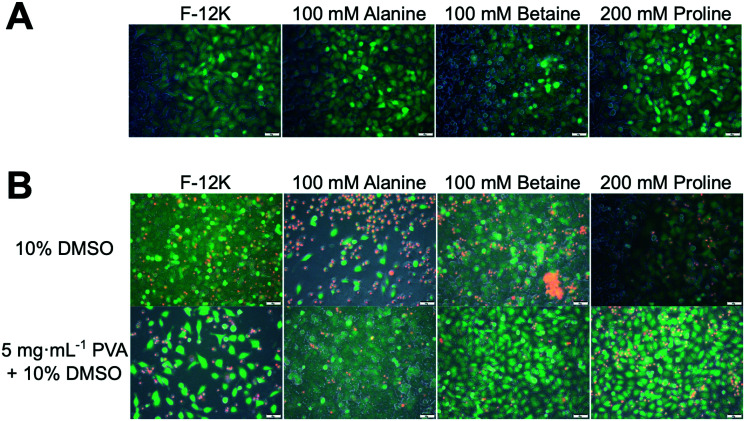
Membrane permeability assays of A549 cells using calcein (green-live)/EthD-1 (red-dead) staining. A) Cells incubated in osmolyte solutions for 24 h then imaged. B) Membrane permeability of frozen/thawed cells which had been previously incubated with indicated osmolyte (or media) and cryopreserved in 10% DMSO (±PVA). Scale bar = 50 μm.

To gain mechanistic insight into the role of proline in cryopreservation, membrane permeabilisation assays were undertaken. Fig. 5A shows results of live/dead staining on A549 cell monolayers after incubation with the osmolytes (green indicates intact membranes, red indicated permeability). In all cases, there was no significant change in membrane permeable (red) cells compared to the controls of media alone (Fig. S6‡). The same assay was undertaken on cryopreserved cells (Fig. 5B) which had been pre-incubated with osmolytes. Minimal differences were seen for cryopreserved cells in any of the osmolyte conditions, with or without PVA, compared to cells frozen with 10% DMSO (Fig. S7‡). This is contrary to the cell recovery data in Fig. 2 and 3, which showed that PVA plus proline or betaine gave higher recovery values than 10% DMSO alone. This suggests that whilst the cryopreserved cells appear to be functionally similar, only cells incubated with proline or betaine and cryopreserved with PVA were able to survive the stress of the subsequent trypsinisation step prior to cell recovery analysis.

**Fig. 6:**
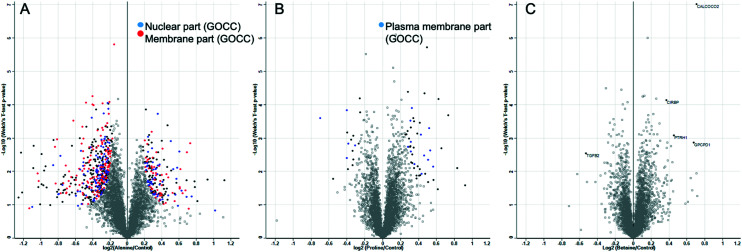
Volcano plots illustrate significantly regulated proteins in A549 cells incubated with A) alanine, B) proline, or C) betaine compared with the control condition. All conditions have been tested in at least quadruplicates. The –log 10 (Welch's *T*-test *P* value) is plotted against the log 2 (fold change as indicated in the *X* axes). The solid black, blue, and red dots represent the significantly regulated proteins (Welch's test, FDR < 0.01 and S0 = 0.1). The dot colours indicate the most abundant enriched gene ontology cellular compartment (GOCC) categories among the significantly regulated proteins according to the Fisher exact test (FDR < 0.0001).

The above data shows that proline has a privileged role in cryopreservation of cell monolayers and acts to slow the growth rate of cells preparing them for cryopreservation without affecting their post-thaw growth rate. This effect is more pronounced than for other osmolytes, and there is no evidence that this is a biophysical process. It is important to again highlight the differential responses seen with other cell lines (Fig. S5‡) to proline pre-incubation. The biochemical mode of action is supported by this differential response and the data presented here does not rule out the use of proline for other cells lines. Further exploration of the large formulation space of concentrations, timings, and cell densities may show a benefit, but highlights that if pre-conditioning (or related strategy) is to be deployed, each line may need individual optimisation. To provide addition insight, whole cell proteomics was undertaken to search for any specific process which might underpin these observations. A549 cells were incubated with the osmolytes for 24 h and then subjected to whole cell proteomic analysis, compared to control cells with the osmolytes. After sample normalisation, 5516 proteins were identified and quantified with a Pearson correlation coefficient >0.99 for all samples (Fig. S8‡). Volcano plots show the distribution of significantly regulated proteins when alanine, proline, and betaine incubated cells were compared against control cells. Gene ontology category Fisher exact test enrichment showed a significant enrichment among the regulated proteins located in the nucleus and membrane when alanine and control conditions were compared. Similar analysis significantly enriched the plasma membrane components when the proline supplemented media was compared to the control (Fig. 6).

Fig. 6 shows that alanine led to more up/down regulation than either proline or betaine, despite resulting in a minimal effect on cryopreservation outcome, compared to the other two osmolytes. Summaries of up/down regulated proteins are shown in the ESI‡ (Fig. S9–S11 and Table S1). For the betaine treated cells, there were 5 significantly different proteins. Upregulation of CALCOCO2 was seen, an effector for clearing bacteria^63^ with implications in cytoskeleton organization,^64^ and upregulation was also seen with proline-incubated cells; CIRBP, a cold-inducible protein implicated in the suppression of cell proliferation;^65^ PTRH1, a t-RNA hydrolase;^66^ and GPCPD1, a glycerophosphocholine phosphodiesterase.^66^ Downregulation was seen for TGFB2, a transforming growth factor beta-2 proprotein, a multifunctional protein that regulates numerous processes. With regard to the proline treated cells, there were no clear single up/down regulated proteins which would allow assignment of the biochemical basis of protection, but there were some regulated proteins which could be important and are summarised here. The upregulation seen for the histone RNA hairpin-binding protein (SLBP) may be due to the increase in HIST1H4A and H1F0 as an increase in histones may result in an increase in the protein involved in the processing, translation, and degradation for the mRNAs of histones.^67^ There was a significant reduction in both SLBP and H1F0 in cells treated with alanine, further suggesting the expression of these proteins is linked. Upregulation in SERPINE1 may provide protection *via* “wound healing” for cells in a monolayer,^68^ as this protein would not be expected to be involved in replicative senescence for A549 adenocarcinoma cells as this process protects against oncogenic transformation and requires integrity of the p53 tumour suppressor pathway.^69–71^ Interestingly, TUT1, a biosensor for the transduction of stress signals within the cell nucleus and also an effector for the fate of target mRNAs,^72^ was shown to be upregulated in proline treated cells but downregulated in cells treated with alanine.

Pathway enrichment analysis for proline showed that general stress responses are the most frequent changes, suggesting this osmolyte has a broad function on cells which could lead to the observed cryopreservation benefits (Table 1). Proline has been implicated as a survival factor that protects the cell against apoptosis and maintains the progression of the cell cycle.^44^ This proteomics study identified the cell nucleus and membranes as the principal cell components affected by our treatments and highlighted some stress responses as potential candidates to explain our previous observations in proline pre-treated cells and their enhanced cryopreservation outcomes. This is not intended as a full description of the cells state, but rather to help guide future research in this area to fully understand the biological impact of this pre-conditioning. This may include focusing on some of the candidates described here to identify a target/effect to further explain proline's function and it may be possible to discover target molecules or pathways to help prepare cells for low temperatures, and hence, increase cell yields.

**Table 1:** Pathway enrichment analysis of significantly regulated proteins in A549 cells incubated with proline

Pathway name	Entities found	Entities total
Apoptosis induced DNA fragmentation	1	13
Cellular responses to stress	8	690
Death receptor signalling	3	157
FGFR2b ligand binding and activation	1	14
TNFR-1-induced proapoptotic signalling	1	14
Chylomicron assembly	1	14
Dissolution of fibrin clot	1	14
Autophagy	3	160

## Conclusions

Here we have explored the role that proline plays in improving the cryopreservation outcomes of cell monolayers, which currently are very challenging to store frozen and result in low cell recovery rates, compared to suspension cryopreservation. The yield of A549 cell monolayers increased from below 25% to ∼50% by supplementation of proline or betaine, which was further increased to 60% by addition of the polymeric ice recrystallization inhibitor PVA. Without the osmolyte pre-incubation, the PVA had no impact. This demonstrates that IRI is useful to enhance cryopreservation, but other (more significant) sources of cryo-damage must be first mitigated. Post-thaw growth rates of proline and betaine treated cells were also closer to non-frozen controls than other conditions, confirming the beneficial role. It was also observed that other cell lines do not respond in the same manner to the proline treatment, suggesting individual optimisation is required. To understand the impact of the pre-freeze incubation, cell growth rates were examined in the presence of osmolytes. It was shown that cell growth rates were significantly suppressed with proline added, but quickly recovered if the media was exchanged, supporting a hypothesis that proline pre-conditions the cells by slowing growth and preparing the cells for cold stress and agrees with observations that some extremophiles upregulate proline biosynthesis in anticipation of cold stress. Whilst betaine also improved cryopreservation outcomes it did not have the same downregulation on cellular growth rates, providing more evidence that the mechanism of action for proline is unique. Membrane stability assays showed that proline did not impact cell membrane permeability compared to control cells or other osmolytes, and biophysical assays of ice formation/growth also confirm that proline had no biophysical role, and hence its function is purely biochemical. Whole cell proteomics were used to explore protein regulation but did not show conclusively any single pathway which could be attributed to this effect. However, when cells were incubated in proline, the upregulation of key histone proteins was observed (which were downregulated in our alanine samples), along with upregulation of stress and membrane proteins, with lipid and proliferation proteins being downregulated, which may be crucial to proline's role but will require further investigation.

Overall this data provides further evidence that pre-conditioning of cell monolayers is a powerful tool to improve post-thaw cell yields, and that proline is an accessible and biocompatible additive that can easily be added into existing protocols. It also suggests that small molecule modulators of cryopreservation could be discovered if the pathways acted upon by proline can be fully elucidated, enabling a chemical biology driven approach towards the discovery of cryoprotective agents/modulators. These findings will help guide processes for improved monolayer storage techniques with subsequent benefits in drug screening, basic research, and improvement for frozen distribution of cells and cell products.

## Author contributions

The manuscript was written through contributions of all authors. All authors have given approval to the final version of the manuscript.

## Conflicts of interest

MG and TB are shareholders in Cryoloyx Ltd.

## Supplementary Material

MD-012-D1MD00078K-s001

## References

[cit1] Healy K. E., McDevitt T. C., Murphy W. L., Nerem R. M. (2013). Engineering the Emergence of Stem Cell Therapeutics. Sci. Transl. Med..

[cit2] Fischbach M. A., Bluestone J. A., Lim W. A. (2013). Cell-Based Therapeutics: The next Pillar of Medicine. Sci. Transl. Med..

[cit3] Stéphenne X., Najimi M., Sokal E. M. (2010). Hepatocyte Cryopreservation: Is It Time to Change the Strategy?. World J. Gastroenterol..

[cit4] Mazur P. (1970). Cryobiology: The Freezing of Biological Systems. Science.

[cit5] Mazur P., Farrant J., Leibo S. P., Chu E. H. (1969). Survival of Hamster Tissue Culture Cells after Freezing and Thawing. Interactions between Protective Solutes and Cooling and Warming Rates. Cryobiology.

[cit6] Timm M., Saaby L., Moesby L., Hansen E. W. (2013). Considerations Regarding Use of Solvents in in Vitro Cell Based Assays. Cytotechnology.

[cit7] Hengstler J. G., Utesch D., Steinberg P., Platt K. L., Diener B., Ringel M., Swales N., Fischer T., Biefang K., Gerl M. (2000). *et al.*, Cryopreserved Primary Hepatocytes as a Constantly Available in Vitro Model for the Evaluation of Human and Animal Drug Metabolism and Enzyme Induction. Drug Metab. Rev..

[cit8] Fahy G. M. (2010). Cryoprotectant Toxicity Neutralization. Cryobiology.

[cit9] Fahy G. M. (1986). The Relevance of Cryoprotectant “Toxicity” to Cryobiology. Cryobiology.

[cit10] Arakawa T., Carpenter J. F., Kita Y. A., Crowe J. H. (1990). The Basis for Toxicity of Certain Cryoprotectants: A Hypothesis. Cryobiology.

[cit11] Da Violante G., Zerrouk N., Richard I., Provot G., Chaumeil J. C., Arnaud P. (2002). Evaluation of the Cytotoxicity Effect of Dimethyl Sulfoxide (DMSO) on Caco2/TC7 Colon Tumor Cell Cultures. Biol. Pharm. Bull..

[cit12] Xu X., Cowley S., Flaim C. J., James W., Seymour L., Cui Z. (2010). The Roles of Apoptotic Pathways in the Low Recovery Rate after Cryopreservation of Dissociated Human Embryonic Stem Cells. Biotechnol. Prog..

[cit13] Boon C. H., Chao P. Y., Liu H., Wei S. T., Rufaihah A. J., Yang Z., Boon H. B., Ge Z., Hog W. O., Eng H. L. (2006). *et al.*, Loss of Viability during Freeze-Thaw of Intact and Adherent Human Embryonic Stem Cells with Conventional Slow-Cooling Protocols Is Predominantly Due to Apoptosis Rather than Cellular Necrosis. J. Biomed. Sci..

[cit14] Bailey T. L., Wang M., Solocinski J., Nathan B. P., Chakraborty N., Menze M. A. (2015). Protective Effects of Osmolytes in Cryopreserving Adherent Neuroblastoma (Neuro-2a) Cells. Cryobiology.

[cit15] Mazur P., Leibo S. P., Chu E. H. Y. A. (1972). Two-Factor Hypothesis of Freezing Injury. Evidence from Chinese Hamster Tissue-Culture Cells. Exp. Cell Res..

[cit16] Fowler A., Toner M. (2005). Cryo-Injury and Biopreservation. Ann. N. Y. Acad. Sci..

[cit17] Mazur P., Principles of Cryobiology, ed. B. Fuller, N. Lane and E. E. Benson, CRC Press, Boca Raton, 2004, vol. 17

[cit18] Mazur P. (1984). Freezing of Living Cells: Mechanisms and Implications. Am. J. Physiol..

[cit19] Mazur P., Miller R. H., Leibo S. P. (1974). Survival of Frozen-Thawed Bovine Red Cells as a Function of the Permeation of Glycerol and Sucrose. J. Membr. Biol..

[cit20] Seth G. (2012). Freezing Mammalian Cells for Production of Biopharmaceuticals. Methods.

[cit21] Chao H., Davies P. L., Carpenter J. F. (1996). Effects of Antifreeze Proteins on Red Blood Cell Survival during Cryopreservation. J. Exp. Biol..

[cit22] Tomás R. M. F., Bailey T. L., Hasan M., Gibson M. I. (2019). Extracellular Antifreeze Protein Significantly Enhances the Cryopreservation of Cell Monolayers. Biomacromolecules.

[cit23] Knight C. A., Hallett J., DeVries A. L. (1988). Solute Effects on Ice Recrystallization: An Assessment Technique. Cryobiology.

[cit24] Knight C. A., Wen D., Laursen R. A. (1995). Nonequilibrium Antifreeze Peptides and the Recrystallization of Ice. Cryobiology.

[cit25] Knight C. A., DeVries A. L., Oolman L. D. (1984). Fish Antifreeze Protein and the Freezing and Recrystallization of Ice. Nature.

[cit26] Carpenter J. F., Hansen T. N. (1992). Antifreeze Protein Modulates Cell Survival during Cryopreservation: Mediation through Influence on Ice Crystal Growth. Proc. Natl. Acad. Sci. U. S. A..

[cit27] Eniade A., Purushotham M., Ben R. N., Wang J. B., Horwath K. A. (2003). Serendipitous Discovery of Antifreeze Protein-Specific Activity in C-Linked Antifreeze Glycoprotein Analogs. Cell Biochem. Biophys..

[cit28] Gibson M. I. M. I. (2010). Slowing the Growth of Ice with Synthetic Macromolecules: Beyond Antifreeze(Glyco) Proteins. Polym. Chem..

[cit29] Inada T., Lu S. S. (2003). Inhibition of Recrystallization of Ice Grains by Adsorption of Poly(Vinyl Alcohol) onto Ice Surfaces. Cryst. Growth Des..

[cit30] Budke C., Koop T. (2006). Ice Recrystallization Inhibition and Molecular Recognition of Ice Faces by Poly(Vinyl Alcohol). ChemPhysChem.

[cit31] Deller R. C. R. C., Vatish M., Mitchell D. A. D. A., Gibson M. I. M. I. (2014). Synthetic Polymers Enable Non-Vitreous Cellular Cryopreservation by Reducing Ice Crystal Growth during Thawing. Nat. Commun..

[cit32] Wowk B., Leitl E., Rasch C. M., Mesbah-Karimi N., Harris S. B., Fahy G. M. (2000). Vitrification Enhancement by Synthetic Ice Blocking Agents. Cryobiology.

[cit33] Deller R. C., Pessin J. E., Vatish M., Mitchell D. A., Gibson M. I. (2016). Enhanced Non-Vitreous Cryopreservation of Immortalized and Primary Cells by Ice-Growth Inhibiting Polymers. Biomater. Sci..

[cit34] Graham B., Bailey T. L., Healey J. R. J., Marcellini M., Deville S., Gibson M. I. (2017). Polyproline as a Minimal Antifreeze Protein Mimic That Enhances the Cryopreservation of Cell Monolayers. Angew. Chem..

[cit35] Qin Q., Zhao L., Liu Z., Liu T., Qu J., Zhang X., Li R., Yan L., Yan J., Jin S. (2020). *et al.*, Bioinspired l -Proline Oligomers for the Cryopreservation of Oocytes via Controlling Ice Growth. ACS Appl. Mater. Interfaces.

[cit36] Burg M. B. (1995). Molecular Basis of Osmotic Regulation. Am. J. Physiol..

[cit37] Yancey P. H., Clark M. E., Hand S. C., Bowlus R. D., Somero G. N. (1982). Living with Water Stress: Evolution of Osmolyte Systems. Science.

[cit38] Higgins C. F., Cairney J., Stirling D. A., Sutherland L., Booth I. R. (1987). Osmotic Regulation of Gene Expression: Ionic Strength as an Intracellular Signal?. Trends Biochem. Sci..

[cit39] Ziyadeh F. N., Mills J. W., Kleinzeller A. (1992). Hypotonicity and Cell Volume Regulation in Shark Rectal Gland: Role of Organic Osmolytes and F-Actin. Am. J. Physiol..

[cit40] Lloyd A. W., Olliff C. J., Rutt K. J. A. (1994). Study of Modified Betaines as Cryoprotective Additives. J. Pharm. Pharmacol..

[cit41] Yang J., Cai N., Zhai H., Zhang J., Zhu Y., Zhang L. (2016). Natural Zwitterionic Betaine Enables Cells to Survive Ultrarapid Cryopreservation. Sci. Rep..

[cit42] Chakrabarti A. C. (1994). Permeability of Membranes to Amino Acids and Modified Amino Acids: Mechanisms Involved in Translocation. Amino Acids.

[cit43] Vilella S., Ahearn G. A., Cassano G., Storelli C. (1988). Na-Dependent L-Proline Transport by Eel Intestinal Brush-Border Membrane Vesicles. Am. J. Physiol..

[cit44] Franěk F., Srámková K. (1997). Cell Suicide in Starving Hybridoma Culture: Survival-Signal Effect of Some Amino Acids. Cytotechnology.

[cit45] Zhang L., Xue X., Yan J., Yan L.-Y., Jin X.-H., Zhu X.-H., He Z.-Z., Liu J., Li R., Qiao J. (2016). Cryobiological Characteristics of L-Proline in Mammalian Oocyte Cryopreservation. Zhonghua Yixue Zazhi.

[cit46] Yang J., Pan C., Zhang J., Sui X., Zhu Y., Wen C., Zhang L. (2017). Exploring the Potential of Biocompatible Osmoprotectants as Highly Efficient Cryoprotectants. ACS Appl. Mater. Interfaces.

[cit47] Koštál V., Šimek P., Zahradníčková H., Cimlová J., Štětina T. (2012). Conversion of the Chill Susceptible Fruit Fly Larva (Drosophila Melanogaster) to a Freeze Tolerant Organism. Proc. Natl. Acad. Sci. U. S. A..

[cit48] Schneider C. A., Rasband W. S., Eliceiri K. (2012). Image to ImageJ: 25 Years of Image Analysis. Nat. Methods.

[cit49] Congdon T., Notman R., Gibson M. I. (2013). Antifreeze (Glyco)Protein Mimetic Behavior of Poly(Vinyl Alcohol): Detailed Structure Ice Recrystallization Inhibition Activity Study. Biomacromolecules.

[cit50] Surís-Valls R., Voets I. K. (2019). The Impact of Salts on the Ice Recrystallization Inhibition Activity of Antifreeze (Glyco)Proteins. Biomolecules.

[cit51] Wu S., Zhu C., He Z., Xue H., Fan Q., Song Y., Francisco J. S., Zeng X. C., Wang J. (2017). Ion-Specific Ice Recrystallization Provides a Facile Approach for the Fabrication of Porous Materials. Nat. Commun..

[cit52] Biggs C. I., Stubbs C., Graham B., Fayter A. E. R., Hasan M., Gibson M. I. (2019). Mimicking the Ice Recrystallization Activity of Biological Antifreezes. When Is a New Polymer “Active”?. Macromol. Biosci..

[cit53] Murthy S. S. N. (1998). Some Insight into the Physical Basis of the Cryoprotective Action of Dimethyl Sulfoxide and Ethylene Glycol. Cryobiology.

[cit54] Murray K. A., Gibson M. I. (2020). Post-Thaw Culture and Measurement of Total Cell Recovery Is Crucial in the Evaluation of New Macromolecular Cryoprotectants. Biomacromolecules.

[cit55] Bailey T. L., Stubbs C., Murray K., Tomas R. M. F., Otten L., Gibson M. I. A. (2019). Synthetically Scalable Poly(Ampholyte) Which Dramatically Enhances Cellular Cryopreservation. Biomacromolecules.

[cit56] Menze M. A., Chakraborty N., Clavenna M., Banerjee M., Liu X. H., Toner M., Hand S. C. (2010). Metabolic Preconditioning of Cells with AICAR-Riboside: Improved Cryopreservation and Cell-Type Specific Impacts on Energetics and Proliferation. Cryobiology.

[cit57] Yamauchi A., Uchida S., Kwon H. M., Preston A. S., Robey R. B., Garcia-Perez A., Burg M. B., Handler J. S. (1992). Cloning of a Na(+)- and Cl(−)-Dependent Betaine Transporter That Is Regulated by Hypertonicity. J. Biol. Chem..

[cit58] Burg M. B., Ferraris J. D., Dmitrieva N. I. (2007). Cellular Response to Hyperosmotic Stresses. Physiol. Rev..

[cit59] Nakanishi T., Balaban R. S., Burg M. B. (1988). Survey of Osmolytes in Renal Cell Lines. Am. J. Physiol..

[cit60] Petronini P. G., De Angelis E. M., Borghetti P., Borghetti A. F., Wheeler K. P. (1992). Modulation by Betaine of Cellular Responses to Osmotic Stress. Biochem. J..

[cit61] Liu W., Hancock C. N., Fischer J. W., Harman M., Phang J. M. (2015). Proline Biosynthesis Augments Tumor Cell Growth and Aerobic Glycolysis: Involvement of Pyridine Nucleotides. Sci. Rep..

[cit62] Hand S., Patil Y., Li S., Charkraborty N., Borcar A., Menze M., Boswell L., Moore D., Toner M. (2013). Diapause and Anhydrobiosis in Embryos of Artemia Franciscana : Metabolic Depression, LEA Proteins and Water Stress. Cryobiology and Cryotechnology.

[cit63] Thurston T. L. M., Wandel M. P., Von Muhlinen N., Foeglein Á., Randow F. (2012). Galectin 8 Targets Damaged Vesicles for Autophagy to Defend Cells against Bacterial Invasion. Nature.

[cit64] Morriswood B., Ryzhakov G., Puri C., Arden S. D., Roberts R., Dendrou C., Kendrick-Jones J., Buss F. (2007). T6BP and NDP52 Are Myosin VI Binding Partners with Potential Roles in Cytokine Signalling and Cell Adhesion. J. Cell Sci..

[cit65] Yang C., Carrier F. (2001). The UV-Inducible RNA-Binding Protein A18 (A18 HnRNP) Plays a Protective Role in the Genotoxic Stress Response. J. Biol. Chem..

[cit66] Gaudet P., Livstone M. S., Lewis S. E., Thomas P. D. (2011). Phylogenetic-Based Propagation of Functional Annotations within the Gene Ontology Consortium. Briefings Bioinf..

[cit67] Marzluff W. F., Wagner E. J., Duronio R. J. (2008). Metabolism and Regulation of Canonical Histone MRNAs: Life without a Poly(A) Tail. Nat. Rev. Genet..

[cit68] Providence K. M., Higgins S. P., Mullen A., Battista A., Samarakoon R., Higgins C. E., Wilkins-Port C. E., Higgins P. J. (2008). SERPINE1 (PAI-1) Is Deposited into Keratinocyte Migration “Trails” and Required for Optimal Monolayer Wound Repair. Arch. Dermatol. Res..

[cit69] Lundberg A. S., Hahn W. C., Gupta P., Weinberg R. A. (2000). Genes Involved in Senescence and Immortalization. Curr. Opin. Cell Biol..

[cit70] Sherr C. J., McCormick F. (2002). The RB and P53 Pathways in Cancer. Cancer Cell.

[cit71] Massagué J. (2004). G1 Cell-Cycle Control and Cancer. Nature.

[cit72] Li W., Laishram R. S., Anderson R. A. (2013). The Novel Poly(A) Polymerase Star-PAP Is a Signal-Regulated Switch at the 3′-End of MRNAs. Adv. Biol. Regul..

