# Supplementary material for: Proline pre-conditioning of cell monolayers increases post-thaw recovery and viability by distinct mechanisms to other osmolytes
Source: RSC Med Chem. 2021 May 18;12(6):982–93. doi: 10.1039/d1md00078k (PMC8221256; doi:10.1039/d1md00078k)
Supplement: MD-012-D1MD00078K-s001 [file MD-012-D1MD00078K-s001.pdf]

Electronic Supplementary Material (ESI) for RSC Medicinal Chemistry.
This journal is © The Royal Society of Chemistry 2021

Supporting Information for

Proline pre-conditioning of cell monolayers increases post-thaw recovery and viability by distinct mechanisms to other osmolytes.

Trisha L. Bailey,[a] Juan Ramon Hernandez-Fernaud,[b] Matthew I. Gibson [a,c]*

[a]Department of Chemistry, University of Warwick, Gibbet Hill Road, Coventry, CV4 7AL, UK

[b]Unidad de Investigación, Hospital Universitario de Canarias. Calle Ofra s/n, La Cuesta, La Laguna, Tenerife, Spain

[c]Warwick Medical School, University of Warwick, Gibbet Hill Road, Coventry, CV4 7AL, UK.

* Corresponding Author m.i.gibson@warwick.ac.uk

Additional Experimental Methods

Cytotoxicity assays. A549 cells were seeded at $4 \cdot 10^4$ cells per well in 200 μL of cell culture medium with indicated concentrations of solutions in 96-well plates (ThermoFisher). Cells were incubated with solutions for 10 min and exchanged against complete cell media or incubated with solutions for 24 h in a humidified atmosphere of 5 % CO_2 and 95 % air at 37 °C. Following the incubation period, resazurin sodium salt (Sigma Aldrich) was dissolved in phosphate buffered saline (Sigma Aldrich) and added to wells in an amount of $1/10^{th}$ initial well volume. Readings were taken using a Synergy HTX Multi-Mode Reader (BioTek, Swindon, UK) at 570/600 nm absorbance every 30 m until control cells reached ~70% reduction. These readings enabled the calculation for the percentage of alamarBlue reduction (Equation 3.1). Values were normalised by dividing experimental values by control values. Cells were then imaged using a CKX41 microscope (Olympus) equipped with the XC30 camera (Olympus). Images were captured and processed using the CellSens standard software (Olympus).

Neuro-2a Cell Culture. Mouse brain neuroblastoma cells (Neuro-2a) were obtained from American Tissue Culture Collection (ATCC) (Middlesex, UK) and grown in 75 cm^2 cell culture Nunc flasks. Standard cell culture medium was composed of Eagle's Minimum Essential Media (EMEM) (Gibco) supplemented with 10 % USA-origin foetal bovine serum (FBS), 100 units$\cdot$mL^{-1} penicillin, 100 μg$\cdot$mL^{-1} streptomycin, and 250 ng$\cdot$mL^{-1} amphotericin B (PSA). Neuro-2a cells were maintained in a humidified atmosphere of 5% CO_2 and 95% air at 37 °C and the culture medium was renewed every 3 - 4 days. The cells were subcultured every 7 days or before reaching 90% confluency. To subculture, cells were dissociated using 0.25%

trypsin plus 1 mM EDTA in balanced salt solution (Gibco) and reseeded at $7.5 \cdot 10^4$ cells per 75 cm^2 cell culture flasks.

MC-3T3 Cell Culture. Mouse calvarial osteoblastic cells (MC-3T3-E1) were obtained from ECACC and grown in 75 cm^2 cell culture Nunc flasks. Standard cell culture medium was composed of Minimum Essential Medium α (MEM-Alpha) (Gibco) supplemented with 10 % USA-origin foetal bovine serum (FBS), 100 units·mL^{-1} penicillin, 100 µg·mL^{-1} streptomycin, and 250 ng·mL^{-1} amphotericin B (PSA). MC-3T3 cells were maintained in a humidified atmosphere of 5% CO_2 and 95% air at 37 °C and the culture medium was renewed every 3–4 days. The cells were subcultured every 7 days or before reaching 90% confluency. To subculture, cells were dissociated using 0.25% trypsin plus 1 mM EDTA in balanced salt solution (Gibco) and reseeded at $1.87 \cdot 10^5$ cells per 75 cm^2 cell culture flasks.

Monolayer plate collagen coating. As indicated in the discussion, to promote attachment of cells, collagen I from rat tail (Sigma-Aldrich) was diluted to 50 µg·mL^{-1} in 200 mM acetic acid (Sigma) and added to each well of a 24-well cell culture plate at 5 µg collagen·cm^{-2}. Plates were incubated with the dissolved collagen for 1 h at room temperature. After this incubation period the collagen solution was removed and the plates were rinsed three times with 200 µL Dulbecco's phosphate buffered saline (Thermo Fisher) to remove any residual acetic acid solution. The collagen treated plates were allowed to dry for 1 h in a laminar flow hood and stored for less than 1 week at 4 °C prior to use.

Proteomics.

Tandem mass tagging proteomics experiments

Sample dissolution and protein digestion. Cell were lysed in 4% SDS, 0.1M Tris-HCl pH7.4, 10 mM TCEP and 40 mM CAA followed by incubation in a preheated thermal block at 95°C for 5 min and bath sonication for 15 min at room temperature. Protein concentration was measured according to manufacturer's instructions using the fluorometric Qubit Protein assay kit (ThermoFisher), measurements were performed using the Qubit Fluorometer (Invitrogen). Aliquots of 300 μg total protein per sample were precipitated methanol-chloroform method.[1] Briefly, experiments were performed at room temperature. Four volumes of methanol were added to one volume of the protein sample, and the mixture was vortexed. One volume of chloroform was then added, and the mixture was vortexed. Three volumes of water was added, and the mixture was vortexed. The sample was centrifuged at 10,000xg for 5 min and the aqueous methanol layer was removed from the top of the sample. The proteins remained at the phase boundary between the aqueous methanol layer and the chloroform layer. Four volumes of methanol were added, and the mixture was vortexed. The sample was spun at 10,000xg for 15 min. The supernatant was removed without disturbing the pellet, and the pellet was air dried. For protein digestion 50 μl of 50 mM HEPES pH8, 60 ng/μl of trypsin (Promega) and 20 ng/μl of LysC (Promega) was added to each sample for overnight proteolysis.

TMT labelling and reverse phase High pH fractionation. After protein digestion, HEPES concentration was adjusted to 200 mM by adding 25 μl of 500 mM HEPES. 25 μg of peptides per each sample was mixed with 0.2 mg of TMT10plex labels (Thermo Scientific) reconstituted in 20 μl of anhydrous acetonitrile. Two set of TMT samples where labelled as follows:

Sample A: 126C, Alanine replicate 1; 127N, Betaine replicate 1; 127C, Betaine replicate 2; 128N, Betaine replicate 3; 128C, Proline replicate 1; 129N, Proline replicate 2; 129C, Proline replicate 3; 130N, Control replicate 2; 130C, Alanine replicate 2; 131N, Alanine replicate 3.

Sample B: 126C, Alanine replicate 1; 127N, Betaine replicate 1; 127C, Betaine replicate 2; 128N, Betaine replicate 3; 128C, Proline replicate 1; 129N, Proline replicate 2; 129C, Proline replicate 3; 130N, Control replicate 2; 130C, Control replicate 1; 131N, Control replicate 3.

After mixing, the samples were incubated for 2 h at room temperature and 1 µg of peptides from each condition was mixed and ran during 2 hours gradient in the mass spectrometer, as described below, to check the labelling efficiency and calculate the coefficient of variance.[2] Samples were kept at -20°C until next step. After, the reaction was quenched with 1 µl of 5% hydroxylamine and 15 µg of peptides per sample were combined for fractionation using High pH Reversed-Phase Peptide Fractionation Kit (Pierce, ThermoFisher) following the manufacturer instructions. All fractions were dried in a vacuum concentrator (Eppendorf) and dissolved in 50 µl of 2% acetonitrile and 0.1% TFA in sonication bath during 5 min.

LC-MS analysis was performed using an Ultimate 3000-RSLCnano system (Dionex) and an Orbitrap-Fusion (Thermo-Scientific). 10 µl (2 µg) of each fraction was loaded on an Acclaim-PepMap µprecolumn (Thermo-Scientific, 300 µm i.d. x 5 mm length, 5 µm particle size, 100 Å pore size) equilibrated in 2 % ACN and 0.1 % TFA, for 6 min at 10 µl/min with an analytical column Acclaim PepMap RSLC (Thermo-Scientific, 75 µm i.d. x 50 cm, 2 µm, 100 Å). Mobile phase A was of 0.1 % formic acid and mobile phase B was ACN containing 0.1 % formic acid. Peptides were eluted at 250 nl/min by increasing the mobile phase B from 5 % B to 22 % over 129 min, then 35 % B over 27 min followed by 80 % B for 3 min and a 15 min re-equilibration at 4 % B. MS data was acquired with Xcalibur v3.0.63 (Thermo-Scientific). Electrospray used a static Nanospray-Flex with a stainless steel emitter OD 1/32' in positive mode at 1.8 kV (Thermo-Scientific). MS survey scans from 375 to 1,500 *m/z*, with a $8x10^5$ ion count target,

maximum injection time of 150 ms and resolution of 60,000 at 200 *m/z*, acquired in profile mode were performed in the Orbitrap analyser. Data dependent mode selected the most abundant precursor ions possible in 3 s cycle time followed by 60 s exclusion and ions were isolated in the quadrupole with a 1 *m/z* window when their intensity was above 40,000. MS/MS scans were performed in the Orbitrap with a $4x10^4$ ion count target, maximum injection time of 500 ms and resolution of 60,000 at 200 *m/z*, acquired in centroid mode. Precursor ions were fragmented with higher energy C-trap dissociation (HCD), normalised collision energy of 38 % and fixed first mass of 120 *m/z*. Thermo-Scientific raw files were analysed using MaxQuant software v1.6.0.16[3] against the UniProtKB Human database (UP000005640, 71,785 entries, release March 2017). Peptide sequences were assigned to MS/MS spectra using the following parameters: cysteine carbamidomethylation as a fixed modification and protein N-terminal acetylation and methionine oxidations as variable modifications. The FDR was set to 0.01 for both proteins and peptides with a minimum length of 7 amino acids and was determined by searching a reversed database. Enzyme specificity was trypsin with a maximum of two missed cleavages. Peptide identification was performed with an initial precursor mass deviation of 7 ppm and a fragment mass deviation of 20 ppm. The MaxQuant feature 'match between runs' was enabled. Reporter ion MS2 and 10plex TMT protein quantification was calculated. Data processing was performed using the Perseus module of MaxQuant v1.6.0.16.[4] Proteins identified by the reverse, contaminant and only by site hits were discarded. Only protein groups identified with at least two assigned peptides were accepted, data was normalised as previously described[2] and intensities were log2 transformed.

Protein groups with significant intensity regulation were determined according to Student's T-test using the Perseus proteomics data analysis tool.[4] Significant hits were filtered using permutation-based FDR <5%. The background proteomics data is available from wrap.warwick.ac.uk.

Additional Data

Phase transitions of individual osmolyte solutions in both PBS, followed by 5 mg·mL^{-1} PVA, and then the combination of PVA + DMSO (Figure S1). This data shows that with the addition of osmolytes or PVA, our solutions are freezing and not vitrifying (i.e. ice formation).

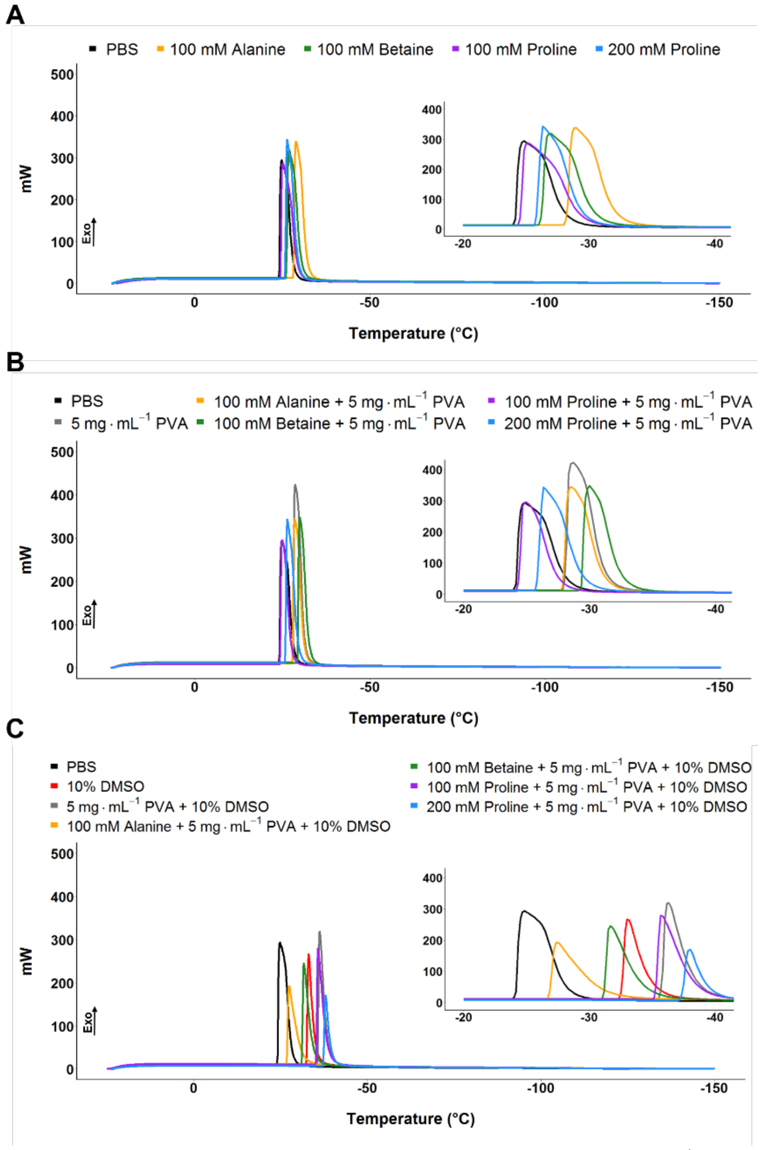

Figure S1. DSC cooling of osmolyte solutions to -150 °C at 10 °C·min^{-1}. **A)** Solutions in PBS. **B)** Solutions with 5 mg·mL^{-1} PVA in PBS. **C)** Solutions with 5 mg·mL^{-1} PVA + 10% DMSO in PBS.

From the heating curves, we can see that we do not have large peak shifts with the addition of osmolytes or PVA (Figure S2).

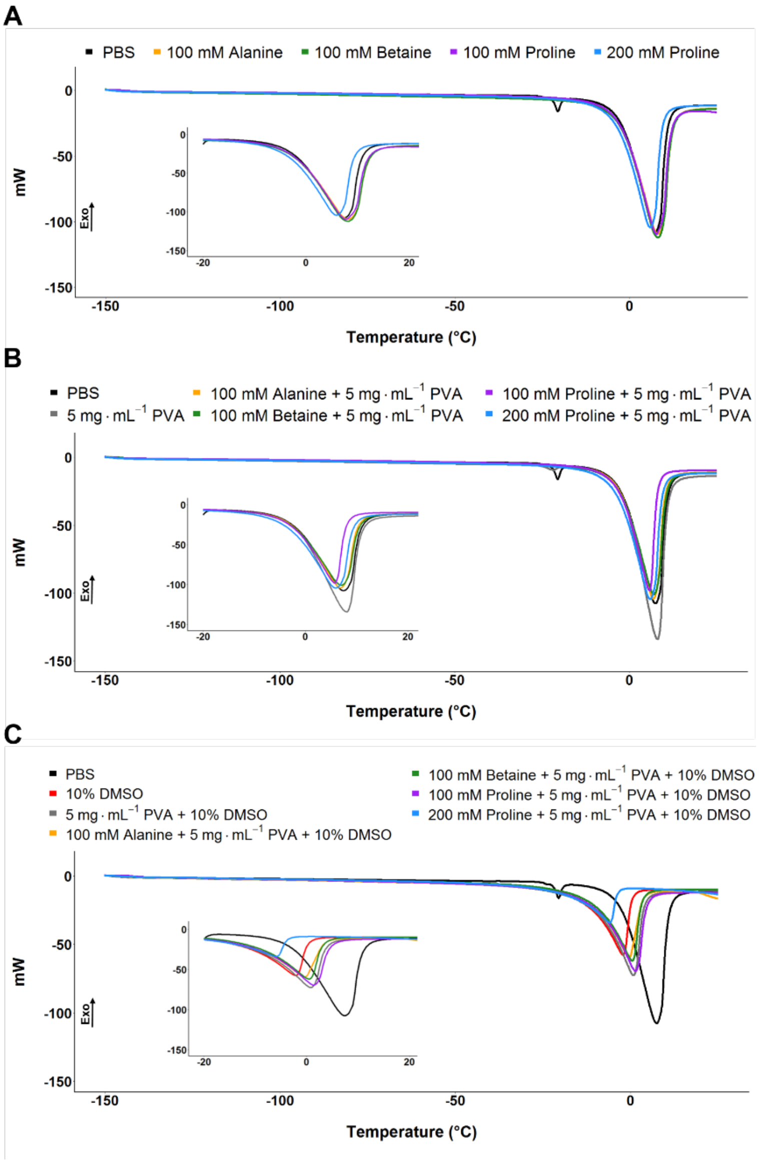

Figure S2. DSC heating of osmolyte solutions warmed from -150 °C to 25 °C at 10 °C·min^{-1}. **A)** Solutions in PBS. **B)** Solutions with 5 mg·mL^{-1} PVA in PBS. **C)** Solutions with 5 mg·mL^{-1} PVA + 10% DMSO in PBS.

We investigated PVA toxicity on A549 cells for 10 min and 24 h and compared these results to cells exposed to 10% DMSO. Normalized alamarBlue reduction for all concentrations of PVA was not significantly different from control cells (0 mM) after a 10 min incubation, however, 10% DMSO did show significantly less reduction following 10 min exposure (Figure S3, $n \geq 3$, P = 0.0000000001). For our 24 h incubation, PVA led to reduced alamarBlue reduction when treated at 1, 2, and 10 mg·mL^{-1}. However, this 24 h PVA reduction was not extreme and was comparable to cells treated with 10% DMSO for 10 min. Cells exposed for 24 h to 10% DMSO had a significantly lower alamarBlue reduction of only 31.9%. This correlates with previous data showing that PVA is non-toxic at up to 20 mg·mL^{-1} concentrations[5] as PVA is regularly utilized in eye drops and is FDA approved. We have shown that PVA was non-toxic for the exposure time of a CPA (10 min) and only minimally reduced metabolic activity when incubated for 24 h (an extreme exposure time).

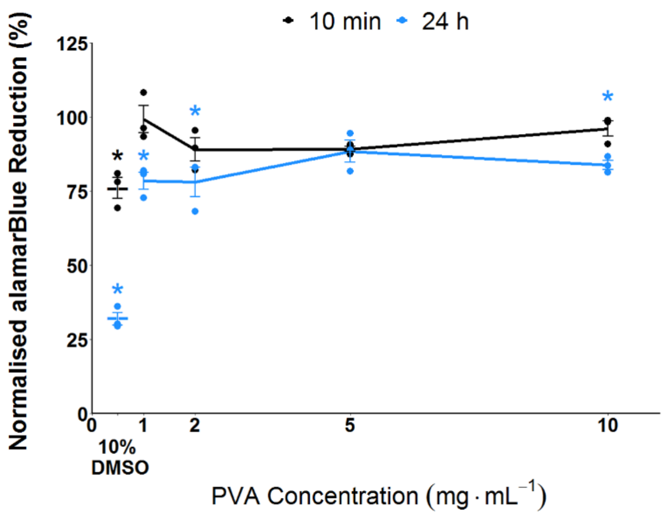

Figure S3. A549 PVA cytotoxicity. Average normalised alamarBlue reduction for 10 min and 24 h. * P < 0.0001 from control (0 mM). Error bars represent ± SEM of at least 3 independent experiments.

AlamarBlue reduction of A549 cells incubated for 24 h with varying concentrations of osmolytes showed no significant difference in reduction compared to control (F-12K 0 mM) (Figure S4, $n = 3$). These results were somewhat unsurprising, as compatible solutes are highly soluble organic compounds of low molecular weight that maintain osmotic balance (osmoregulation) without interfering with cell metabolism.[6] We have shown that incubation with osmolytes for 24 h did not alter the metabolic capabilities of the cells compared to control and were non-toxic to our cell monolayers.

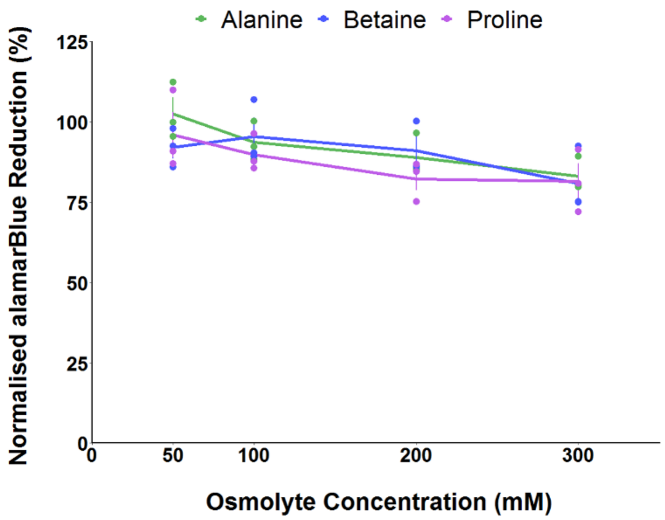

Figure S4. A549 osmolyte cytotoxicity**.** Average normalised alamarBlue reduction after 24 h incubation. Error bars represent ± SEM of 3 independent experiments.

Encouraged by the post-thaw increase in the number of recovered cells, proline was tested using two additional cell lines; mouse calvarial osteoblastic (MC-3T3) and mouse brain neuroblastoma (Neuro-2a). There was some improvement in post-freeze recovery for MC-3T3 cells when proline and PVA were used, but this recovery was not significantly different from cells frozen with only 10% DMSO on either bare plates (Supplementary Figure 5A) or collagen coated plates (Supplementary Figure 5B). MC-3T3 cells are a contact inhibited cell line, so

growth arrest may be triggered by simply creating a confluent monolayer. It is important to note that MC-3T3 cells are considerably larger (20–50 μm)[7] than A549 cells (~15 μm)[8] and hence intracellular ice formation may be more likely in MC-3T3 cells. There was no significant improvement for the monolayer freezing of Neuro-2a cells incubated in 200 mM proline on collagen coated plates (Supplementary Figure 5C). While our Neuro-2a cells were similar in size (10-14 μm)[9] to our A549 cells, neuronal cells are very sensitive to hypotonic conditions[10] so these cells may be much more sensitive to the harsh event of cryopreservation, thus a higher concentration of proline may be needed. There was, however, a trend that high concentrations of PVA did increase recovery in a non-linear fashion. PVA is not easily soluble past 20 $mg \cdot mL^{-1}$ so could not be tested further.

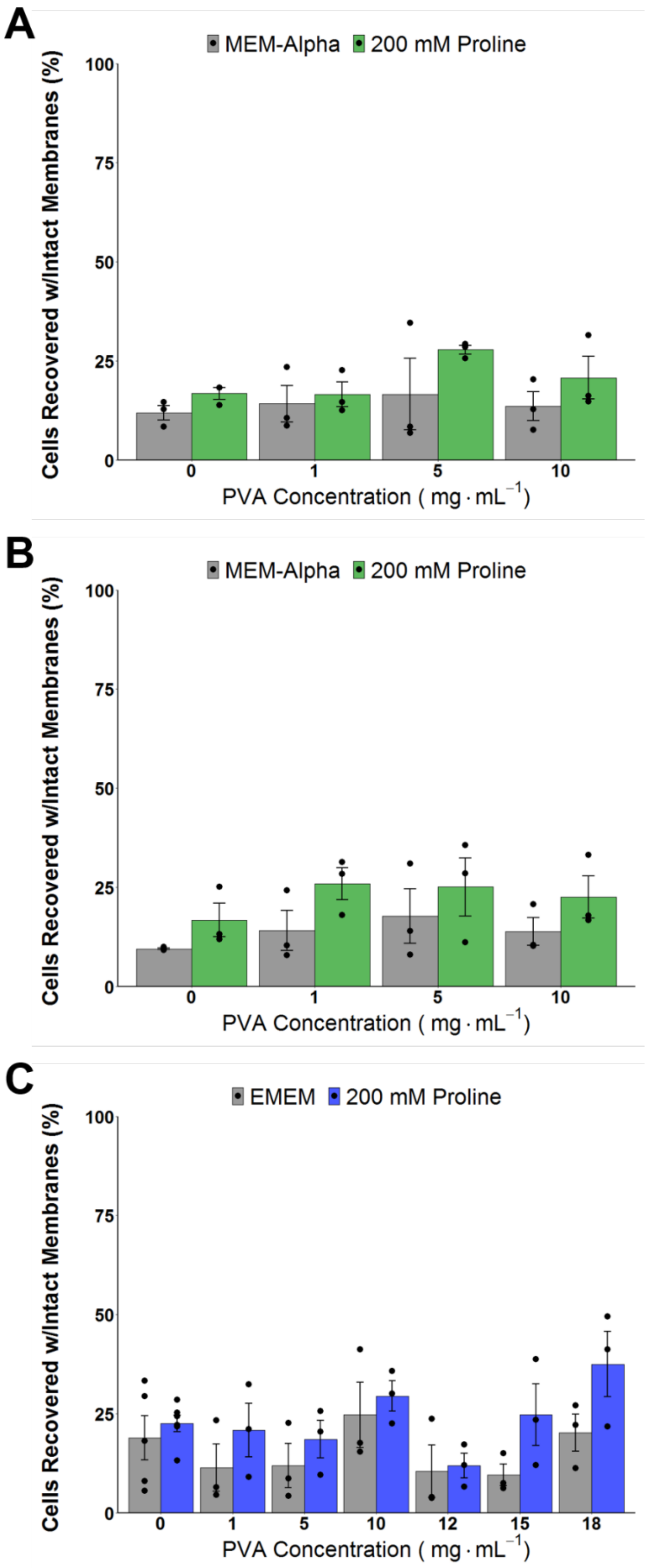

Figure S5. Immortalized cell line PVA concentration monolayer freezing. Cells recovered after freezing to -80 °C. **A)** MC-3T3 cells frozen on bare plate. **B)** MC-3T3 cells frozen on

collagen coated plates. **C)** Neuro-2a cells frozen on collagen coated plates. Error bars represent ± SEM of 3 independent experiments.

There was no significant difference in calcein fluorescence (green) for cells incubated in osmolyte solutions (Supplementary Figure 6A) and cells incubated in 200 mM proline had significantly lower EthD-1 fluorescence (red) compared to 100 mM alanine and 100 mM betaine (Supplementary Figure 6B, n = 3, P = 0.02) but all conditions were comparable to control cells. Incubating cells in osmolyte solutions for 24 h did not significantly alter the permeability of the cells relative to control cells.

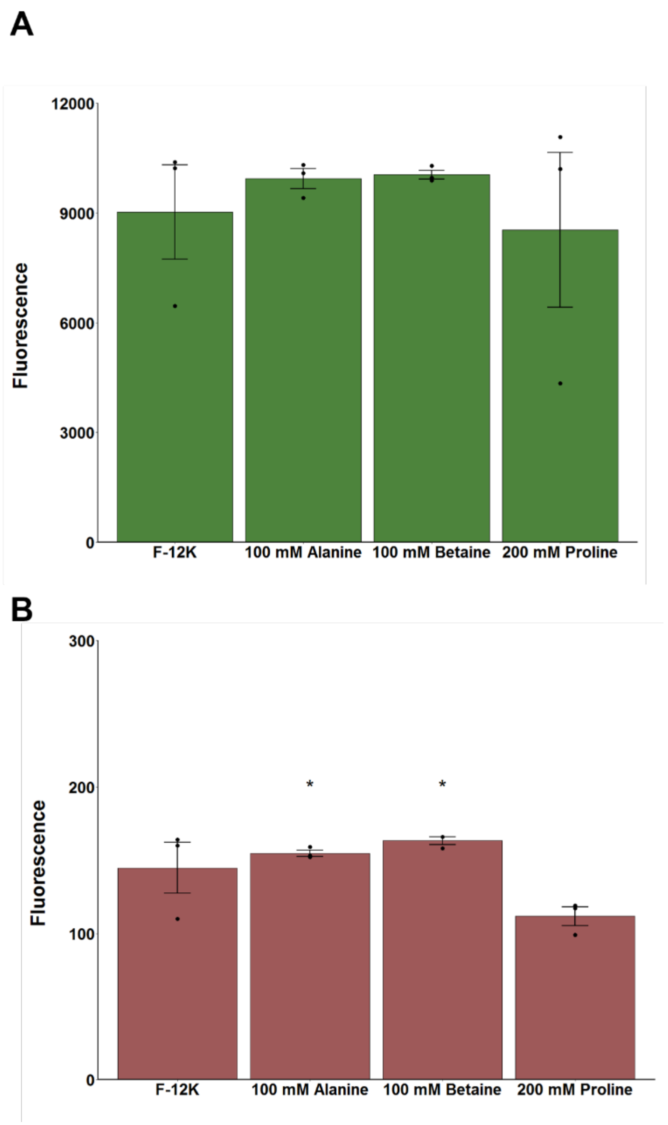

Figure S6. Calcein (494Ex/517Em) / EthD-1 (517Ex/617Em) fluorescent readings of A549 cells incubated in osmolyte solutions for 24 h. **A)** Calcein readings. **B)** Ethidium homodimer-1 readings. * $P < 0.03$ from 200 mM proline. Error bars represent ± SEM of 3 independent experiments.

Cells were incubated in osmolyte pre-treatments for 24 h (as indicated) then treated with the specified CPA for 10 min, frozen for 24 h to -80 °C, thawed with 37 °C cell media (F-12K) and incubated for 24 h, then treated with calcein-AM and EthD-1. Cells frozen with osmolyte pre-incubation and with or without PVA did not significantly differ in their calcein AM or

EthD-1 uptake from 10% DMSO, with the exception of 100 mM alanine which had significantly higher EthD-1 fluorescence (Supplementary Figure 7, $n = 3$, $P = 0.00006$).

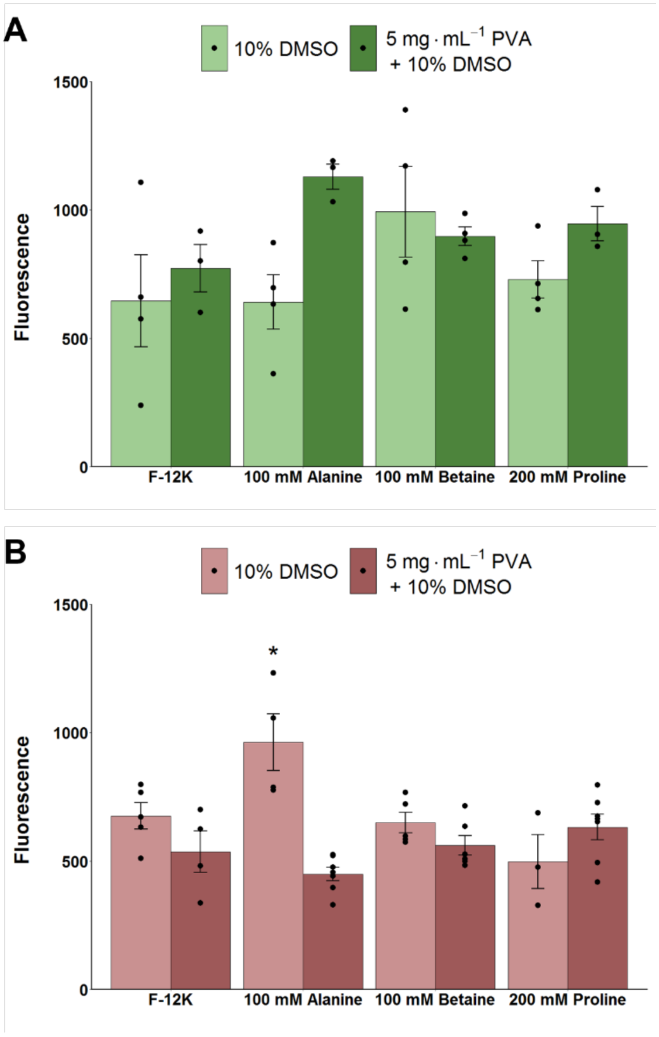

Figure S7. Calcein (494Ex/517Em) / EthD-1 (517Ex/617Em) fluorescent readings of frozen/thawed A549 cells with osmolyte and PVA treatments. **A)** Calcein readings. **B)** Ethidium homodimer-1 readings. * $P < 0.001$ from 10% DMSO in F-12K.

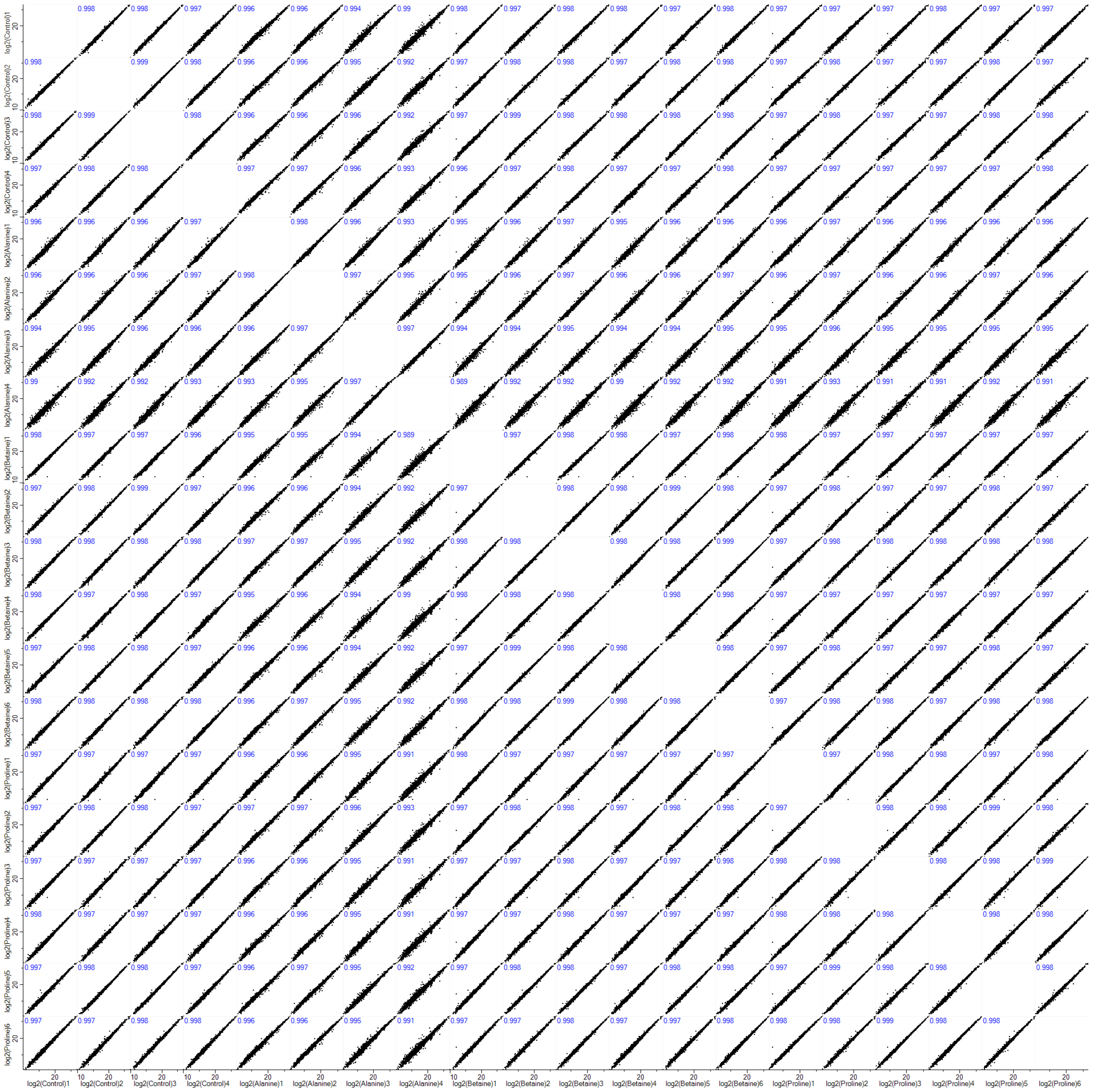

Figure S8. Comparison of all control, alanine, betaine, and proline proteomic samples against each other, measured at least in quadruplicates. The matrix of correlation plots reveals correlations with Pearson correlation coefficient > 0.99 for all samples.

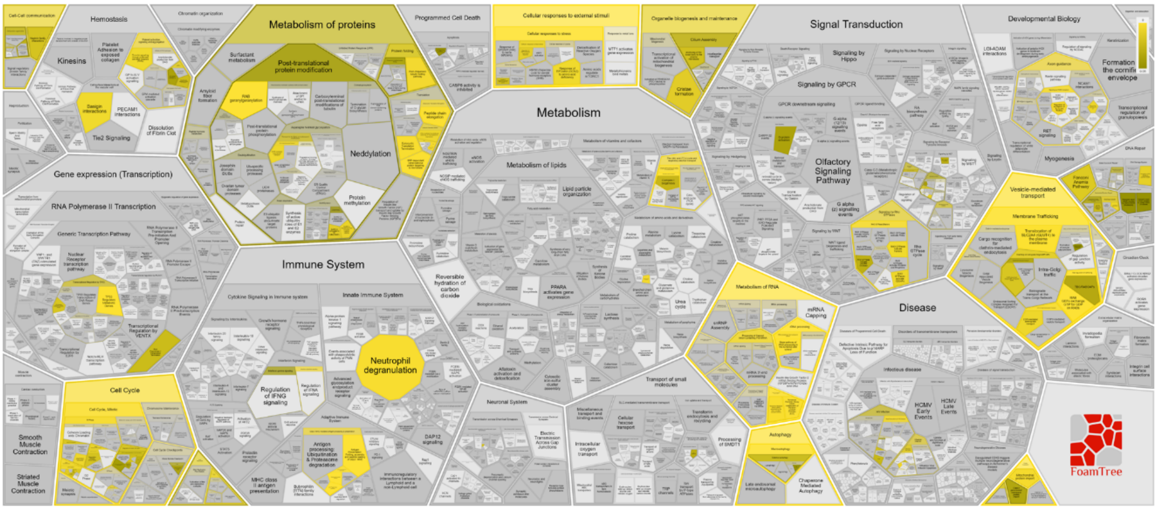

Figure S9. Pathway enrichment analysis foam tree diagram of significantly regulated proteins in A549 cells incubated with alanine. Data analysed online with Reactome open access pathway database.[11] Coloured in yellow are shown the significantly enriched pathways (FDR>0.01). The reactome pathway knowledgebase. https://reactome.org/

Table S1. Pathway enrichment analysis of significantly regulated proteins in A549 cells incubated with alanine. Data analysed online with Reactome open access pathway database.

Pathway name	Entities found	Entities total	Entities FDR
Endosomal/Vacuolar pathway	22	82	2.70E-05
Antigen Presentation: Folding, assembly and peptide loading of class I MHC	22	102	5.21E-04
Membrane Trafficking	74	665	5.21E-04
Antigen processing-Cross presentation	30	187	1.22E-03
ER-Phagosome pathway	27	165	2.12E-03
Rab regulation of trafficking	22	129	6.64E-03
Respiratory electron transport, ATP synthesis by chemiosmotic coupling, and heat production by uncoupling proteins.	23	146	1.23E-02
Cellular responses to stress	69	690	1.23E-02
Metabolism of RNA	76	782	1.23E-02
Protein localization	25	170	1.24E-02
RAB GEFs exchange GTP for GDP on RABs	17	94	1.46E-02
Respiratory electron transport	19	115	1.70E-02
Cellular responses to external stimuli	69	708	1.70E-02
Interferon alpha/beta signaling	25	184	2.89E-02
Macroautophagy	21	144	3.32E-02
rRNA processing	30	245	3.32E-02
Major pathway of rRNA processing in the nucleolus and cytosol	25	189	3.32E-02
RAB geranylgeranylation	13	68	3.32E-02
Translocation of SLC2A4 (GLUT4) to the plasma membrane	14	79	3.80E-02
Autophagy	22	160	3.80E-02
Host Interactions of HIV factors	21	150	3.80E-02
Vesicle-mediated transport	75	824	3.80E-02
The citric acid (TCA) cycle and respiratory electron transport	28	229	4.05E-02
rRNA processing in the nucleus and cytosol	26	207	4.07E-02

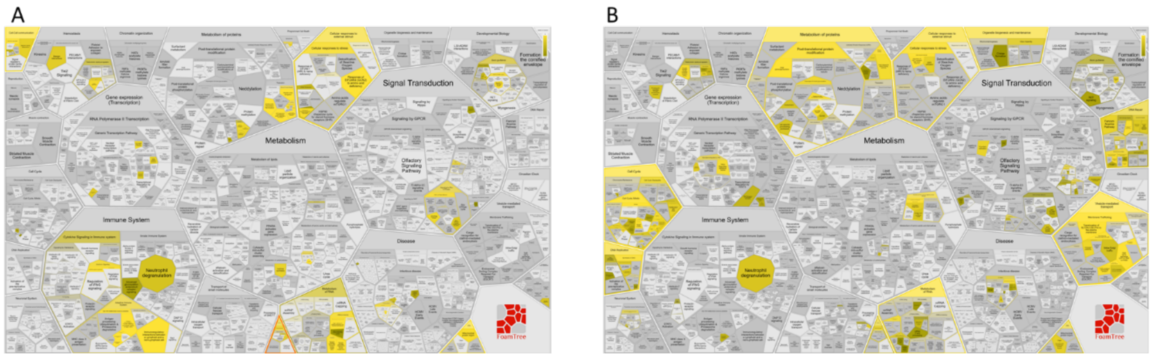

Figure S10. Pathway enrichment analysis foam tree diagram of significantly A) up-regulated and B) down-regulated proteins in A549 cells incubated with alanine. Data analysed online with Reactome open access pathway database.[11] Coloured in yellow are shown the significantly enriched pathways (FDR>0.01).

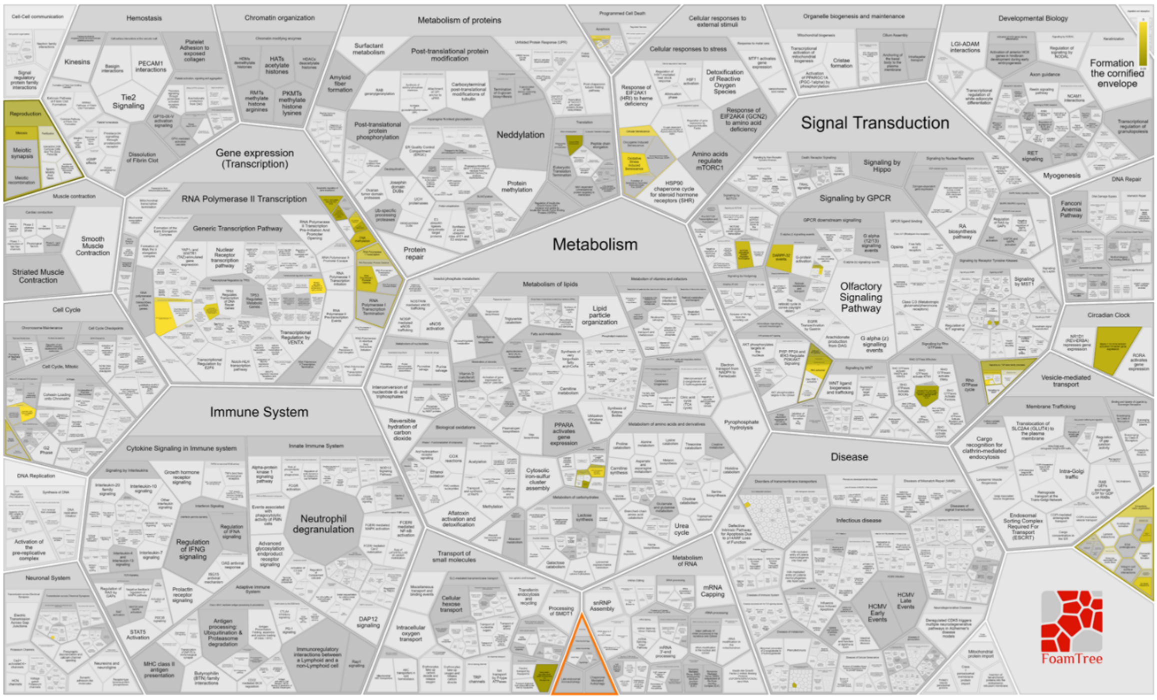

Figure S11. Pathway enrichment analysis foam tree diagram of significantly regulated proteins in A549 cells incubated with proline. Data analysed online with Reactome open access pathway database.[11] Coloured in yellow are shown the significantly enriched pathways (FDR>0.01).

References

(1) Fic, E.; Kedracka-Krok, S.; Jankowska, U.; Pirog, A.; Dziedzicka-Wasylewska, M. Comparison of Protein Precipitation Methods for Various Rat Brain Structures Prior to Proteomic Analysis. *Electrophoresis* **2010**, *31* (21), 3573–3579. https://doi.org/10.1002/elps.201000197.

(2) Plubell, D. L.; Wilmarth, P. A.; Zhao, Y.; Fenton, A. M.; Minnier, J.; Reddy, A. P.; Klimek, J.; Yang, X.; David, L. L.; Pamir, N. Extended Multiplexing of Tandem Mass Tags (TMT) Labeling Reveals Age and High Fat Diet Specific Proteome Changes in Mouse Epididymal Adipose Tissue. *Mol. Cell. Proteomics* **2017**, *16* (5), 873–890. https://doi.org/10.1074/mcp.M116.065524.

(3) Tyanova, S.; Temu, T.; Cox, J. The MaxQuant Computational Platform for Mass Spectrometry-Based Shotgun Proteomics. *Nat. Protoc.* **2016**, *11* (12), 2301–2319. https://doi.org/10.1038/nprot.2016.136.

(4) Tyanova, S.; Temu, T.; Sinitcyn, P.; Carlson, A.; Hein, M. Y.; Geiger, T.; Mann, M.; Cox, J. The Perseus Computational Platform for Comprehensive Analysis of (Prote)Omics Data. *Nat. Methods* **2016**, *13* (9), 731–740. https://doi.org/10.1038/nmeth.3901.

(5) Deller, R. C.; Pessin, J. E.; Vatish, M.; Mitchell, D. A.; Gibson, M. I. Enhanced Non-Vitreous Cryopreservation of Immortalized and Primary Cells by Ice-Growth Inhibiting Polymers. *Biomater. Sci.* **2016**, *4* (7), 1079–1084. https://doi.org/10.1039/C6BM00129G.

(6) Julca, I.; Alaminos, M.; González-López, J.; Manzanera, M. Xeroprotectants for the Stabilization of Biomaterials. *Biotechnol. Adv.* **2012**, *30* (6), 1641–1654. https://doi.org/10.1016/j.biotechadv.2012.07.002.

(7) Sudo, H.; Kodama, H. A.; Amagai, Y.; Yamamoto, S.; Kasai, S. In Vitro Differentiation and Calcification in a New Clonal Osteogenic Cell Line Derived from Newborn Mouse Calvaria. *J. Cell Biol.* **1983**, *96* (1), 191–198. https://doi.org/10.1083/jcb.96.1.191.

(8) Jiang, R.; Shen, H.; Piao, Y.-J. The Morphometrical Analysis on the Ultrastructure of A549 Cells. *Rom. J. Morphol. Embryol.* **2010**, *51* (4), 663–667.

(9) Zalis, M. C.; Reyes, J. F.; Augustsson, P.; Holmqvist, S.; Roybon, L.; Laurell, T.; Deierborg, T. Label-Free Concentration of Viable Neurons, HESCs and Cancer Cells by Means of Acoustophoresis. *Integr. Biol. (Camb).* **2016**, *8* (3), 332–340. https://doi.org/10.1039/c5ib00288e.

(10) Carpaneto, A.; Accardi, A.; Pisciotta, M.; Gambale, F. Chloride Channels Activated by Hypotonicity in N2A Neuroblastoma Cell Line. *Exp. brain Res.* **1999**, *124* (2), 193–199. https://doi.org/10.1007/s002210050614.

(11) Jassal, B.; Matthews, L.; Viteri, G.; Gong, C.; Lorente, P.; Fabregat, A.; Sidiropoulos, K.; Cook, J.; Gillespie, M.; Haw, R.; Loney, F.; May, B.; Milacic, M.; Rothfels, K.; Sevilla, C.; Shamovsky, V.; Shorser, S.; Varusai, T.; Weiser, J.; et al. The Reactome Pathway Knowledgebase. *Nucleic Acids Res.* **2020**, *48* (D1), D498–D503. https://doi.org/10.1093/nar/gkz1031.
